# Plasma metabolites associated with type 2 diabetes in a Swedish population: a case–control study nested in a prospective cohort

**DOI:** 10.1007/s00125-017-4521-y

**Published:** 2018-01-18

**Authors:** Lin Shi, Carl Brunius, Marko Lehtonen, Seppo Auriola, Ingvar A. Bergdahl, Olov Rolandsson, Kati Hanhineva, Rikard Landberg

**Affiliations:** 1 Department of Molecular Sciences, Swedish University of Agricultural Sciences, Uppsala, Sweden; 2 Department of Biology and Biological Engineering, Chalmers University of Technology, Gothenburg, Sweden; 3 School of Pharmacy, University of Eastern Finland, Kuopio, Finland; 4 LC-MS Metabolomics Center, Biocenter Kuopio, Kuopio, Finland; 5 Department of Biobank Research, Umeå University, Umeå, Sweden; 6 Department of Public Health and Clinical Medicine, Umeå University, Umeå, Sweden; 7 Institute of Public Health and Clinical Nutrition, Department of Clinical Nutrition, University of Eastern Finland, Kuopio, Finland; 8 Unit of Nutritional Epidemiology, Institute of Environmental Medicine, Karolinska Institute, Stockholm, Sweden; 9 Present Address: Department of Biology and Biological Engeneering, Food and Nutrition Science, Chalmers University of Technology, SE-412 96 Gothenburg, Sweden

**Keywords:** Metabolomics, Multivariate modelling, Predictive biomarker, Reproducibility, Risk prediction, Traditional risk factor, Type 2 diabetes

## Abstract

**Aims/hypothesis:**

The aims of the present work were to identify plasma metabolites that predict future type 2 diabetes, to investigate the changes in identified metabolites among individuals who later did or did not develop type 2 diabetes over time, and to assess the extent to which inclusion of predictive metabolites could improve risk prediction.

**Methods:**

We established a nested case–control study within the Swedish prospective population-based Västerbotten Intervention Programme cohort. Using untargeted liquid chromatography-MS metabolomics, we analysed plasma samples from 503 case–control pairs at baseline (a median time of 7 years prior to diagnosis) and samples from a subset of 187 case–control pairs at 10 years of follow-up. Discriminative metabolites between cases and controls at baseline were optimally selected using a multivariate data analysis pipeline adapted for large-scale metabolomics. Conditional logistic regression was used to assess associations between discriminative metabolites and future type 2 diabetes, adjusting for several known risk factors. Reproducibility of identified metabolites was estimated by intra-class correlation over the 10 year period among the subset of healthy participants; their systematic changes over time in relation to diagnosis among those who developed type 2 diabetes were investigated using mixed models. Risk prediction performance of models made from different predictors was evaluated using area under the receiver operating characteristic curve, discrimination improvement index and net reclassification index.

**Results:**

We identified 46 predictive plasma metabolites of type 2 diabetes. Among novel findings, phosphatidylcholines (PCs) containing odd-chain fatty acids (C19:1 and C17:0) and 2-hydroxyethanesulfonate were associated with the likelihood of developing type 2 diabetes; we also confirmed previously identified predictive biomarkers. Identified metabolites strongly correlated with insulin resistance and/or beta cell dysfunction. Of 46 identified metabolites, 26 showed intermediate to high reproducibility among healthy individuals. Moreover, PCs with odd-chain fatty acids, branched-chain amino acids, 3-methyl-2-oxovaleric acid and glutamate changed over time along with disease progression among diabetes cases. Importantly, we found that a combination of five of the most robustly predictive metabolites significantly improved risk prediction if added to models with an a priori defined set of traditional risk factors, but only a marginal improvement was achieved when using models based on optimally selected traditional risk factors.

**Conclusions/interpretation:**

Predictive metabolites may improve understanding of the pathophysiology of type 2 diabetes and reflect disease progression, but they provide limited incremental value in risk prediction beyond optimal use of traditional risk factors.

**Electronic supplementary material:**

The online version of this article (10.1007/s00125-017-4521-y) contains peer-reviewed but unedited supplementary material, which is available to authorised users.


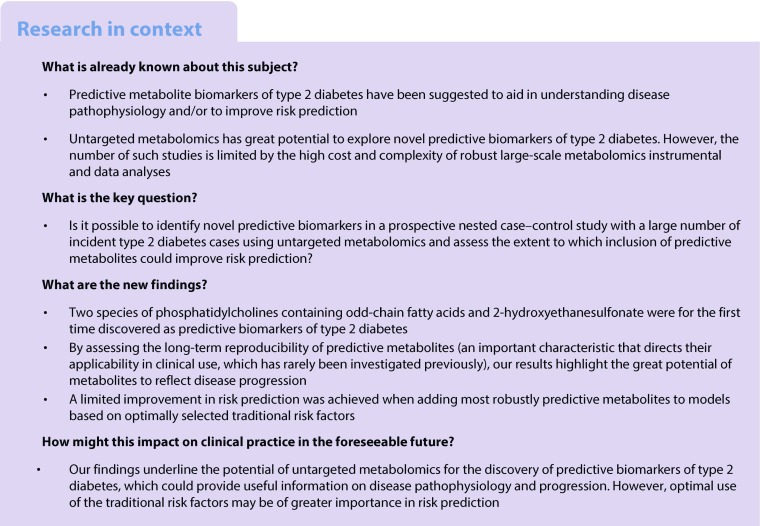


## Introduction

Type 2 diabetes is a metabolic disorder characterised by insulin resistance in target tissues and deficiency of insulin secretion in the pancreas [1]. Identification of individuals at risk of developing type 2 diabetes is particularly important for prevention and early intervention [2]. Metabolomics is an emerging tool to discover metabolic alterations before onset of disease, thereby potentially providing novel insights into disease pathophysiology and/or improving disease prediction [2, 3]. Early alterations in branched-chain amino acids (BCAAs), phospholipids and acylcarnitines have been linked to type 2 diabetes risk, but the complex metabolic alterations underlying the pathophysiology remain unclear [4].

Untargeted liquid chromatography (LC)-MS metabolomics applied in prospective cohort studies covers more metabolites and may have greater potential to capture the overall metabolic status, provide more comprehensive information for mechanistic investigations and improve prediction [5]. Recent studies applying untargeted LC-MS metabolomics have successfully uncovered novel metabolites associated with future type 2 diabetes [6–11]. However, studies in general have some limitations, e.g. small number of incident diabetes cases [6, 8, 10], lack of generalisability due to single-sex cohorts [6, 9], limited analytical platform set-up [7–9], and ambiguous metabolite annotation by only matching molecular mass against online databases [7, 8]. Such limitations may partly explain some of the inconsistent findings across studies.

In most observational studies, metabolites have been determined in a single sample at baseline [12, 13]. However, metabolites are typically subject to both random and systematic variations over time and random intra-individual variability of a single measurement will affect the precision in risk estimates when linking a potential biomarker to endpoint and bias any observed association towards null [14]. Therefore, the reproducibility of predictive metabolites for type 2 diabetes is an important characteristic that directs the applicability in epidemiological and clinical investigations but has rarely been investigated before.

Several metabolomics studies aiming to develop novel biomarkers for type 2 diabetes have included discovered predictive metabolites in combination with traditional risk factors, i.e. anthropometry and/or biochemical measures in disease risk prediction models, and have found improved risk prediction [4, 8, 15–18]. However, in all these studies potential metabolite predictors have been added to a priori defined models based on established risk scores and/or a subset of risk factors. To our knowledge, no study has added metabolites to risk factors that were optimally selected to fit the cohort in order to elucidate the independent contribution of metabolites in predicting type 2 diabetes risk.

We therefore established a nested case–control study within the Swedish Västerbotten Intervention Programme cohort [19] to identify predictive plasma metabolites of type 2 diabetes, using untargeted LC-MS metabolomics. We also assessed the association of these metabolites with insulin resistance and beta cell function, to investigate their role in glucose homeostasis. Moreover, we evaluated the long-term reproducibility and systematic changes in these metabolites from baseline up to 10 years of follow-up among controls and cases, respectively. Furthermore, we assessed the extent to which inclusion of metabolites beyond traditional risk factors could improve risk prediction, using different variable selection methods. To our knowledge, this study constitutes the hitherto largest untargeted metabolite-profiling study of incident type 2 diabetes in a nested case–control setting.

## Methods

### Study population

**Fig. 1:**
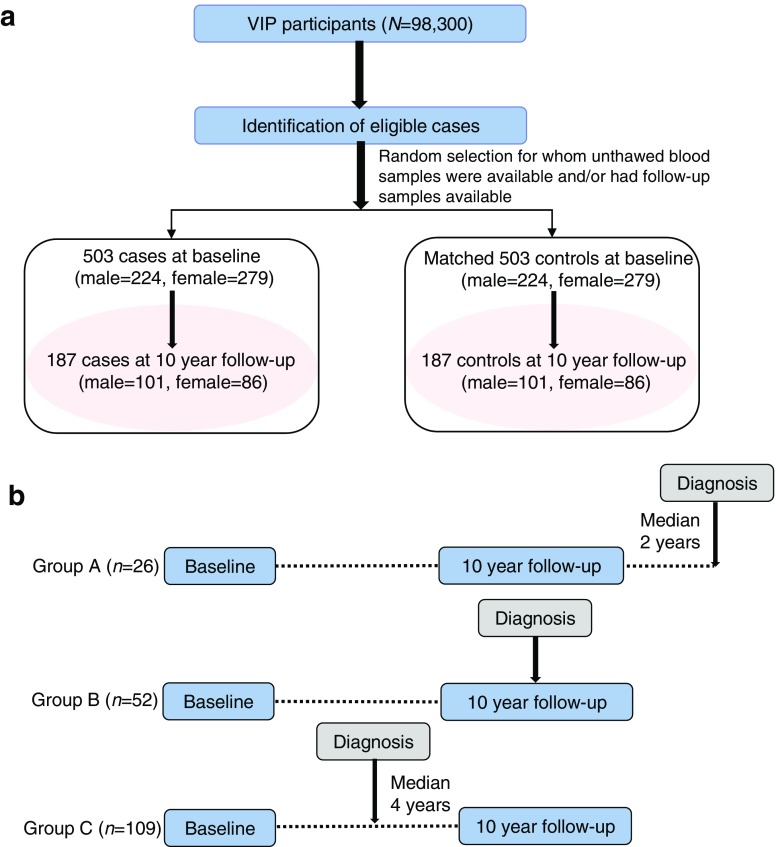
(**a**) Flowchart of participant selection from the Västerbotten Intervention Programme cohort. (**b**) Information on baseline and 10 year follow-up sampling among 187 type 2 diabetes cases. Group A, where the second sample was drawn before (median 2 years) type 2 diabetes diagnosis (*n* = 26 pairs); group B, where the second sample was drawn in the same year of type 2 diabetes diagnosis (*n* = 52 pairs); group C, where the second sample was drawn after (median 4 years) type 2 diabetes diagnosis (*n* = 109 pairs)

The investigation was set up as a case–control study nested within the Västerbotten Intervention Programme cohort [19]. Details of the cohort can be found in the electronic supplementary material (ESM) Methods. Among 3256 incident diabetes cases identified from the diabetes registry DiabNorth [20], we selected 503 participants at baseline who had an unthawed fasting plasma sample in the biobank and who later developed type 2 diabetes after a median time of 7 years (Fig. 1a). Each case was individually matched to one non-diabetic individual according to age (±2 years), sex, ethnic group and season of blood draw. Among the 503 pairs of selected participants, 187 case–control pairs had a second follow-up sample drawn and data collected 10 years after baseline (Fig. 1b). The corresponding characteristics of the subgroup of 187 case–control pairs with follow-up data were similar to those of the 503 case–control pairs and those without available repeated samples (ESM Table 1). To investigate changes in metabolites over time in relation to the time of diagnosis, we then assigned the 187 pairs of participants to three groups, depending on when the diagnosis was made in relation to the second sample (ESM Table 2): group A, where the second sample was drawn before (median 2 years) diagnosis (*n* = 26 pairs); group B, where the second sample was drawn in the same year as diagnosis (*n* = 52 pairs); and group C, where the second sample was drawn after (median 4 years) diagnosis (*n* = 109 pairs). In addition, to examine the effect of medication on metabolite levels during follow-up, we stratified the 187 cases into four groups: no medication (*n* = 19), only glucose-lowering medication (*n* = 13), other medication (*n* = 48), and glucose-lowering and other medication (*n* = 107) (ESM Table 3). Informed consent was obtained from all participants included in the prospective cohort study from which biological samples analysed in this study originated. The study protocol was approved by the regional ethics committee in Uppsala, Sweden (registration number 2014/011).

### Untargeted LC-MS metabolomics

Details of the technical procedures are described elsewhere [21] and are provided in ESM Methods. In brief, fasting heparin plasma samples were de-proteinised and analysed by HPLC-qTOF-MS/MS (Agilent QTOF 6540, Agilent Technologies, Santa Clara, CA USA). Reverse-phase and hydrophilic interaction chromatography were applied to detect both lipophilic and hydrophilic metabolites, using both positive and negative electrospray ionisation modes. Plasma samples were analysed in eight batches; randomisation was constrained to keep sample pairs and follow-up samples within the same batch, but otherwise there was full randomisation within batch. The stability and functionality of the system were monitored throughout all the instrumental analyses using quality control samples.

### Data preprocessing

Throughout this article, the term ‘feature’ refers to a mass spectral peak, i.e. a molecular entity with a unique mass-to-charge ratio and retention time (RT) as measured by an LC-MS instrument. The term ‘metabolite’ refers to a metabolite, with or without successful identification.

Briefly, data deconvolution was performed with XCMS (www.bioconductor.org; downloaded in March 2016) using parameters obtained from an optimisation procedure (ESM Methods, ESM Fig. 1, ESM Table 4). Within- and between-batch correction for instrumental drift in RT, mass accuracy and signal intensity was performed [21]. Qualified features potentially generated from a single metabolite were aggregated based on PUTMEDID-LCMS (http://www.mcisb.org/resources/putmedid.html; downloaded in May 2016) [22], to reduce the over-representation of a particular metabolite. In total, 29,240 features were retained after a stringent normalisation procedure (ESM Methods, ESM Fig. 1). Missing values were replaced with random selection from a normal distribution between zero and the lowest measured peak intensity within the feature.

### Statistical analysis

#### Discovery of metabolites that predicted future type 2 diabetes

A comprehensive data analysis pipeline was applied to identify predictive metabolites of type 2 diabetes (ESM Fig. 1). Briefly, sparse partial least squares regression was performed as a pre-filter on data obtained from each chromatograph to remove the majority of uninformative features that were unlikely to contribute to discrimination between cases and controls. Pre-filtered data were then processed using an in-house developed multilevel partial least squares (ML-PLS) classification algorithm. We incorporated standard ML-PLS [23] into a repeated double cross-validation framework with unbiased variable selection, which effectively determines a parsimonious set of discriminative features ranked according to their importance with reduced risk of statistical overfitting [24, 25]. The predictive ability of the constructed models outperformed those of 500 permuted models, demonstrating robustness and validity of the ML-PLS model in discriminating cases from controls (ESM Fig. 2). Following ML-PLS analysis, 2743 of the 29,240 qualified features were selected, of which 79 features were top-ranked (i.e. where the variable importance ranking score was <100, with a lower score indicating a high importance of a given discriminative feature between cases and controls) in reverse-phase data and 92 were top-ranked in hydrophilic interaction chromatography data. Top-ranked features had the highest importance for discrimination; including more features did not substantially improve the discrimination (data not shown). Features that were annotated as ‘metabolites’ (see ‘Identification of metabolites’ below) were subjected to subsequent analyses.

Conditional logistic regression was applied to calculate the OR of type 2 diabetes with metabolites at baseline using R package survival [26]. For each metabolite, ORs were calculated for quartiles and per SD increment. A crude model was calculated for each metabolite. To investigate whether associations were independent from known risk factors, we constructed: model 1, adjusting for fasting plasma glucose (FPG, mmol/l) and BMI, (kg/m^2^); model 2, further adjusting for physical activity (inactive, moderately inactive, moderately active, active), education (elementary school, vocational school, secondary school, university education/college), smoking (smoker, former smoker, occasional smoker, non-smoker), and consumption of alcohol (sex-specific, g/day), dietary fibre (g/day), red and processed meat (g/day) and coffee (g/day); and model 3, further adjusting for total cholesterol (mmol/l), triacylglycerols (mmol/l) and systolic and diastolic BP (mmHg). We also assessed the association between metabolites and incident diabetes cases after excluding 95 case participants who had FPG ≥5.9 mmol/l and 2 h plasma glucose (2 h-PG) >11.1 mmol/l at baseline, or who developed diabetes during the first 2 years after baseline sampling. To compensate for multiple testing, false discovery rate-adjusted *p* values were calculated; the significance threshold was set at *p <* 0.05.

#### Assessment of changes in predictive metabolites over time

Reproducibility of metabolites among controls was estimated by intra-class correlation (ICC) over the 10 year period between the two sampling occasions among the subset of healthy participants (*n* = 187), using an SAS macro (%ICC9; SAS Institute, Cary, NC, USA). If the mean metabolite concentration between occasions differed significantly, ICC was instead calculated on rank-transformed data. We also performed mixed models to investigate changes in metabolites over the 10 year period in relation to the time of diagnosis among cases. In a secondary analysis, paired *t* tests were applied to examine whether metabolite levels differed between baseline and follow-up among cases, stratified by medication.

#### Evaluation of the predictive performance of metabolites

We assessed whether metabolites could improve risk prediction using two approaches: (1) by adding predictive metabolites to covariates used in model 1 or model 2 (this approach has been used in most published studies [4, 8, 15–18]); or (2) through a selection of optimal variables from traditional risk factors and/or metabolites using a validated random forest algorithm [24]. This unbiased variable selection approach resulted in three models with an optimal number of the most relevant predictors based on maximised prediction performance and minimised risk of statistical overfitting [24]. For models based on the second approach above, the metabolite score was based only on selected variables from the annotated predictive metabolites (Metabolomics Standard Initiative [MSI] 1–2), the traditional risk score (TS) was based on 14 known traditional type 2 diabetes risk factors to which we had access (age, FPG, BMI, 2 h-PG, total cholesterol, triacylglycerols, systolic- and diastolic BP, consumption of coffee, dietary fibre, red and processed meat, and education, physical activity and smoking), and the combined score (CS) was based on optimal variable selection among both metabolites and traditional risk factors. All scores were calculated according to the method described previously [8]. 2 h-PG is a widely accepted cornerstone of prediabetes diagnostics, but it is rarely applied in large cohort studies due to time and cost. Therefore, we repeated the selection approach for TS-_2h-PG_, excluding 2 h-PG from the list of variables. The area under the receiver operating characteristic (AUC_ROC_) was computed using R package pROC [27] to evaluate prediction performance of different models. To avoid overfitting, we randomly split the samples 10,000 times into training (60%) and test sets (40%) for prediction and validation. The mean of AUC_ROC_ values was calculated from 10,000 ROC curves and the 95% CIs were calculated as the 2.5 and 97.5 percentile values. We used Wilcoxon’s signed-rank test to determine differences in the predictive performance between different models. Moreover, we also assessed the incremental predictive performance of metabolite score by using the net reclassification improvement and integrated discrimination improvement test using R package PredictABEL [28] .

#### Correlations

Spearman correlation coefficients were calculated to explore the association of metabolites with traditional risk factors at baseline among 503 healthy participants. Partial Spearman correlations were calculated to investigate independent associations between each of the metabolites and HOMA-IR and HOMA-derived beta cell function (HOMA-%B), adjusted for BMI, age, sex and case–control status among 187 case–control pairs with follow-up samples. HOMA-IR and HOMA-%B were computed using the HOMA calculator (www.dtu.ox.ac.uk; accessed 1 June 2017).

### Identification of metabolites

We identified metabolites based on accurate mass and product ion spectrum matching against an in-house library of authentic standards, online databases and literature (ESM Table 5). The confidence level of annotation was categorised according to the MSI reporting criteria [29]. Lipids were verified in both positive and negative electrospray ionisation modes according to their characteristic product ions. The annotated classes (MSI 3) are presented as ‘chemical class mass@RT’, while unknown compounds (MSI 4) are presented as ‘mass@RT’.

## Results

Baseline characteristics of the 503 pairs of participants are presented in Table 1. Several known type 2 diabetes risk factors were higher in cases than in controls. BMI and HOMA-IR were higher in both cases and controls at 10 year follow-up compared with baseline, while total cholesterol and HOMA-%B were lower in cases at follow-up.

**Table 1:** Baseline characteristics of participants who later developed type 2 diabetes and their matched controls in a case–control study nested within the Västerbotten Intervention Programme cohort

Characteristic	Cases (*n* = 503)	Matched controls (*n* = 503)	*p* value for difference
Men^a^	44.5	44.5	
Age, years^a^	50.2 (7.9)	50.1 (8.0)	
Fasting glucose, mmol/l	6.0 (0.9)	5.5 (1.1)	<0.0001
2 h-PG, mmol/l	8.3 (2.8)	6.5 (1.6)	<0.0001
BMI, kg/m^2^	29.5 (4.9)	25.5 (3.8)	<0.0001
HOMA-IR^b^	1.7 (1.1)	0.9 (0.7)	<0.0001
HOMA-%B^b^	101.5 (72.9)	74.5 (27.7)	<0.0001
Triacylglycerols, mmol/l	2.0 (1.3)	1.4 (0.7)	<0.0001
Total cholesterol, mmol/l	5.9 (1.2)	5.7 (1.1)	<0.0001
Systolic BP, mmHg	138 (18.1)	128 (17.2)	<0.0001
Diastolic BP, mmHg	85 (10.4)	80 (9.7)	<0.0001
Total energy intake, kJ/day	7186.0 (2551.2)	7282.2 (2594.1)	0.6
Dietary fibre, g/day	18.9 (7.4)	19.5 (8.2)	0.3
Whole grains, g/day	72.2 (36.5)	74.4 (39.6)	0.5
Fat, g/day	68.2 (26.0)	64.3 (27.7)	0.6
Alcohol, g/day	3.3 (6.7)	3.6 (4.3)	0.1
Smoking status			0.03
Current smoker	22.4	19.1	
Former smoker	28.6	25.8	
Occasional smoker	1.0	3.8	
Former occasional smoker	9.2	7.2	
Non-smoker	38.7	43.9	
Physical activity^c^			0.1
Inactive	18.7	17.9	
Moderately inactive	35.4	35.9	
Moderately active	28.2	27.3	
Active	17.7	18.9	
Education			0.04
Elementary school	33.3	29.8	
Vocational (training) school	28.1	26.2	
Secondary school	22.3	20.6	
University education/college	16.3	23.4	

Data are mean (SD) or %

^a^Matching factors

^b^HOMA-IR and HOMA-%B at baseline among a subset of 187 case–control pairs with repeated samples available

^c^Physical activity defined based on the Cambridge physical activity index [47], which is a validated index based on two questions in the Västerbotten Intervention Programme questionnaire related to physical activity in work and leisure

**Fig. 2:**
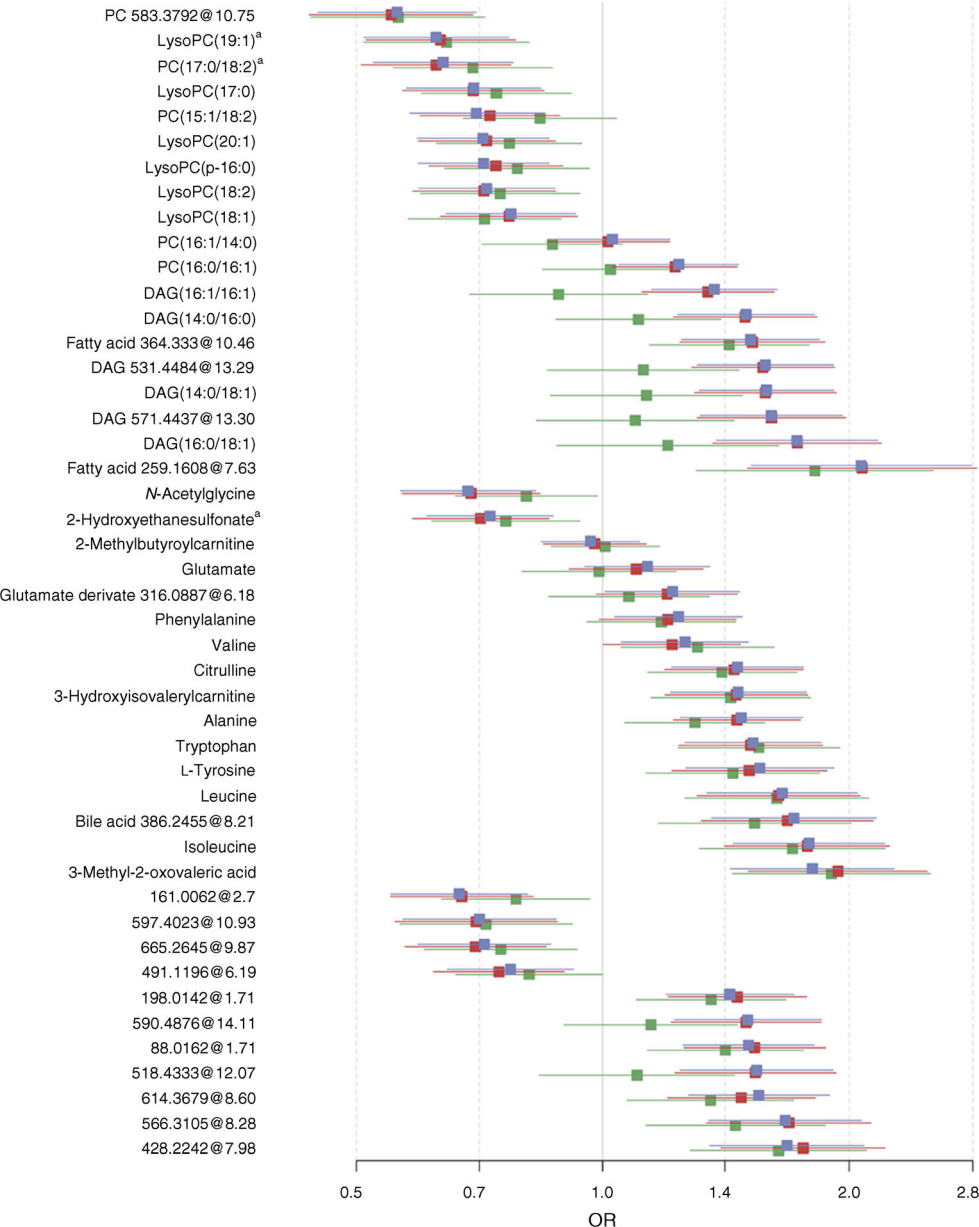
ORs per SD increment (95% CI) of metabolites based on results from multivariate-adjusted conditional logistic regression models. Model 1 (blue): adjustment for FPG, BMI; model 2 (red): further adjustment for physical activity, education, smoking, consumptions of alcohol, dietary fibre, red and processed meat and coffee intake; model 3 (green): additional adjustment for plasma total cholesterol, triacylglycerols, and systolic and diastolic BP. Error bars indicate the 95% CI; ^a^ denote novel predictive biomarkers found in the current study

We discovered 46 predictive metabolites of type 2 diabetes, including novel findings, i.e. phosphatidylcholines (PCs) containing odd-chain fatty acids (C19:1 and C17:0) and 2-hydroxyethanesulfonate, as well as previously identified predictive biomarkers and 11 unknowns (Fig. 2, Table 2, ESM Table 6). Of these 46 metabolites, 44 were associated with ORs of type 2 diabetes independently of baseline BMI and FPG, and 42 remained significant after further adjustment for lifestyle factors. On further adjustment for total cholesterol, triacylglycerols and BP, associations were overall attenuated, but 33 metabolites remained significant (Fig. 2, ESM Table 6). Exclusion of cases with abnormal glucose levels or cases who developed diabetes during the first 2 years after baseline sampling did not substantially affect results (ESM Fig. 3).

**Table 2:** Metabolites that were significantly associated with odds of developing type 2 diabetes in the present study and in studies reported in the literature, metabolite changes at the 10 year follow-up among controls and cases, and effect of medication in the present study

MetaMetabolite	Association with risk of developing type 2 diabetes at baseline			Changes in metabolites over time			Medication
	Direction^a^	Pathophysiology^b^	References^c^	ICC (95% CI)^d^	Cases vs controls^e^	Baseline vs follow-up^f^	
LysoPC(18:2)	–	HOMA-IR	[6, 10, 15]	0.38 (0.26, 0.50)	Lower		
LysoPC(18:1)	–	HOMA-IR	[10, 15]	0.51 (0.40, 0.61)	Lower		
LysoPC(p-16:0)	–	HOMA-IR		0.42 (0.31, 0.48)	Lower	Lower	
LysoPC(17:0)	–	HOMA-IR	[10, 30]	0.22 (0.11, 0.38)	Lower	Lower	
LysoPC(19:1)	–	HOMA-IR, HOMA-%B		0.5 (0.40, 0.61)	Lower	Lower	
LysoPC(20:1)	–	HOMA-IR	[10]	0.27 (0.17, 0.41)	Lower		
PC(16:0/16:1)	+	HOMA-IR	[48]	0.25 (0.14, 0.40)	Higher		Affected^g^
PC 583.3792@10.75	–	HOMA-IR		0.10 (0.03, 0.31)	Lower		
PC(16:1/14:0)^h^	+			0.25 (0.14, 0.40)	Higher		
PC(15:1/18:2)^h^	–	HOMA-IR, HOMA-%B	[10]	0.45 (0.34, 0.56)	Lower	Lower	
PC(17:0/18:2)	–	HOMA-IR		0.21 (0.11, 0.38)	Lower		
DAG(16:1/16:1)^h^	+	HOMA-IR, HOMA-%B		0.28 (0.16, 0.42)	Higher		Affected^g^
DAG(14:0/16:0)^h^	+	HOMA-IR, HOMA-%B		0.31 (0.20, 0.45)	Higher		Affected^g^
DAG 531.4484@13.29^h^	+	HOMA-IR, HOMA-%B		0.36 (0.25, 0.49)	Higher		Affected^g^
DAG 571.4437@13.30^h^	+	HOMA-IR, HOMA-%B		0.40 (0.29, 0.53)	Higher		Affected^g^
DAG(14:0/18:1)^h^	+	HOMA-IR, HOMA-%B		0.37 (0.25, 0.49)	Higher		Affected^g^
DAG(16:0/18:1)^h^	+	HOMA-IR, HOMA-%B		0.43 (0.32, 0.55)	Higher		Affected^g^
Fatty acid 364.333@10.46	+	HOMA-IR^i^		0.35 (0.24, 0.48)	Higher		
Fatty acid 259.1608@7.63	+	HOMA-IR, HOMA-%B		0.49 (0.38, 0.59)	Higher		
2-Methylbutyroylcarnitine	+	HOMA-IR^i^	[9, 39]	0.54 (0.44, 0.64)	Higher		
3-Hydroxyisovalerylcarnitine	+		[9, 39]	0.41 (0.30, 0.53)	Higher		
Phenylalanine	+	HOMA-IR^i^	[11, 38]	0.62 (0.53, 0.70)	Higher		Affected^g^
Leucine	+	HOMA-IR, HOMA-%B	[10, 15, 38]	0.65 (0.57, 0.73)	Higher	Higher	
Isoleucine	+	HOMA-IR, HOMA-%B	[10, 15, 38]	0.68 (0.59, 0.75)	Higher	Higher	
Valine	+	HOMA-IR, HOMA-%B	[10, 15, 16, 38]	0.50 (0.37, 0.58)	Higher	Higher	
Tryptophan	+	HOMA-IR, HOMA-B%^i^	[48]	0.46 (0.35, 0.57)	Higher		Affected^g^
l-Tyrosine	+	HOMA-IR	[9, 11, 38]	0.50 (0.39, 0.60)	Higher		
Alanine	+		[11, 49]	0.32 (0.21, 0.45)	Higher		
Citrulline	+	HOMA-IR^i^	[40]	0.43 (0.32, 0.54)	–		
*N*-Acetylglycine^h^	–	HOMA-IR, HOMA-%B^i^	[6]	0.39 (0.28, 0.51)	Lower		Affected^g^
2-Hydroxyethanesulfonate	–	HOMA-IR, HOMA-%B^i^		0.34 (0.23, 0.47)	Lower		Affected^g^
Glutamate	+	HOMA-IR, HOMA-%B	[49]	0.39 (0.28, 0.52)	Higher	Higher	
Glutamate derivate 316.0887@6.19	+	HOMA-IR, HOMA-%B		0.41 (0.30, 0.53)	Higher	Higher	
Bile acid386.2455 @8.21	+	HOMA-IR, HOMA-%B		0.65 (0.56, 0.72)	Higher	Higher	
3-Methyl-2-oxovaleric acid	+	HOMA-IR^i^	[6, 39]	0.61 (0.48, 0.67)	Higher	Higher	
161.0062@2.7	–	HOMA-IR, HOMA-%B		0.45 (0.35, 0.57)	Lower		Affected^g^
88.0162@1.71	+	HOMA-IR		0.23 (0.12, 0.38)	Higher		
198.0142 @1.71	+	HOMA-IR		0.24 (0.13, 0.40)	Higher		
491.1196@6.19	–	HOMA-IR		0.52 (0.42, 0.62)	Lower		
518.4333@12.07	+	HOMA-IR, HOMA-%B		0.40 (0.28, 0.52)	Higher		
590.4876@14.11	+	HOMA-IR, HOMA-%B		0.35 (0.24, 0.48)	Higher		
428.2242@7.98	+	HOMA-IR, HOMA-%B		0.76 (0.70, 0.82)	Higher		Affected^g^
566.3105@8.28	+	HOMA-IR, HOMA-%B		0.54 (0.44, 0.64)	Higher		
614.3679@8.60	+			0.63 (0.54, 0.71)	Higher		
665.2645@9.87	–			0.50 (0.40, 0.61)	Lower		
597.4023@10.93	–	HOMA-IR^i^		0.22 (0.12, 0.38)	Lower		Affected^g^

^a^Direction: + denotes a higher concentration of metabolite present in cases, while − denotes a lower concentration of metabolites compared with cases

^b^Metabolites at baseline correlated significantly (Bonferroni-adjusted *p <* 0.05) with HOMA-IR and/or HOMA-%B

^c^Previous findings reported in the literature from 2013 to the present. For each metabolite, the list of papers is not exhaustive. Reviews are not considered. For lipids, reference is made only to publications that report fatty acid constituents

^d^ICC represents long-term reproducibility of metabolites among healthy controls (*n* = 187) over 10 years. ICC ≥0.4 denotes good to excellent reproducibility

^e^Difference between cases (*n* = 187) and their matched controls, independent of BMI, age, sex and time to diagnosis stratification. ‘Higher’ means metabolite level is higher in the case than in the matched control, and vice versa

^f^The difference between baseline and the 10 year follow-up among cases, independent of BMI, age, sex and time to diagnosis stratification. ‘Higher’ means metabolite level is higher at follow-up than at baseline, and vice versa

^g^Group-specific difference in metabolites between baseline and 10 year follow-up in 187 cases. Four groups were created according to medication: no medication (*n* = 19), only glucose-lowering medication (*n* = 13), other medication (*n* = 48), and glucose-lowering and other medication (*n* = 107). ‘Affected’ means that medication affected changes in metabolite levels between baseline and 10 year follow-up among cases

^h^Group-specific differences in metabolites between baseline and 10 year follow-up in 187 cases. Three groups were created depending on when type 2 diabetes diagnosis occurred in relation to the 10 year follow-up (group A: repeated sampling before diagnosis; group B: repeated sampling close to diagnosis; group C: repeated sampling after diagnosis)

^i^Partial Spearman correlations of metabolites with HOMA-%B and/or HOMA-IR were affected by time to diagnosis, and significant correlations were only found a median time of 6 years before type 2 diabetes onset

Among 46 identified metabolites, 26 showed intermediate to high reproducibility among healthy controls (0.4 ≤ ICC ≤ 0.75). Moreover, lysoPC(p-16:0), lysoPC(19:1) and PC(15:1/18:2) were inversely associated with type 2 diabetes at baseline and were lower among cases at the 10 year follow-up compared with baseline, regardless of whether the second sample was taken before, at the same time or after diagnosis. The opposite was found for BCAAs, 3-methyl-2-oxovaleric acid, bile acid and glutamate, all of which were increased at follow-up (Table 2, ESM Fig. 4). In a secondary analysis, we found that changes in diglycerides (DAGs), phenylalanine, tryptophan, *N*-acetylglycine and 2-hydroxyethanesulfonate between baseline and follow-up differed across cases with or without different medications (Table 2, ESM Table 7).

**Fig. 3:**
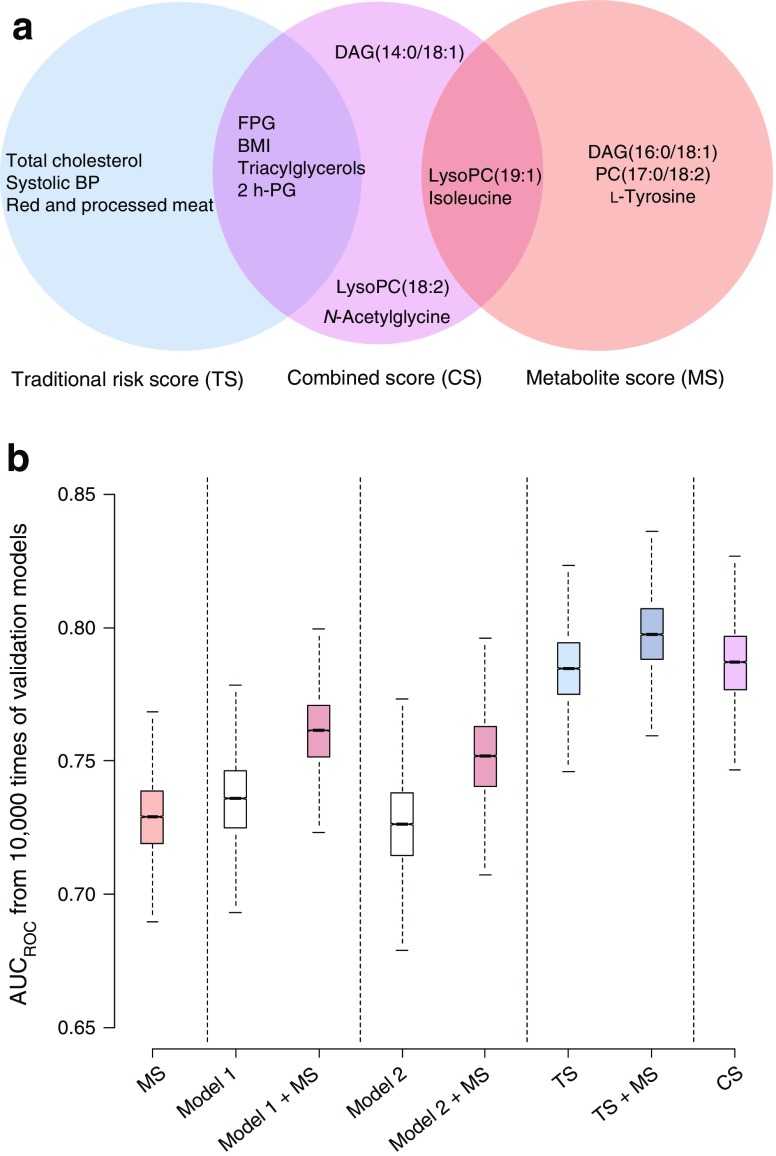
Comparison of the prediction performance of clinical risk factors, metabolites and their combinations for risk of type 2 diabetes. (**a**) Optimally selected subset of predictors, employing a validated random forest algorithm, for TS, CS and metabolite score (MS). (**b**) Prediction performance of different models trained from metabolites, traditional risk factors and their combinations. AUC_ROC_ values were obtained from 10,000 models where the samples were randomly split into training (60%) and test sets (40%) for prediction and validation; the AUC_ROC_ values were 0.73 (95% CI 0.69, 0.76) for MS, 0.74 (95% CI 0.70, 0.77) for model 1, 0.77 (95% CI 0.73, 0.79) for model 1 + MS, 0.72 (95% CI 0.67, 0.74) for model 2, 0.75 (95% CI 0.72, 0.78) for model 2 + MS, 0.78 (95% CI 0.76, 0.81) for TS, 0.80 (95% CI 0.77, 0.83) for TS + MS and 0.79 (95% CI 0.76, 0.82) for CS. Adding an MS to model 1 resulted in a continuous net reclassification improvement (NRI) of 0.85 (95% CI 0.73, 0.95) and an integrated discrimination improvement (IDI) of 0.16 (95% CI 0.14, 0.19) (*p* < 0.001 for both analyses), and to model 2 NRI 0.76 (95% CI 0.65, 0.88) and IDI 0.12 (0.09, 0.14) (*p* < 0.01 for both analyses), indicating a significant improvement in risk stratification. Adding MS to TS resulted in a marginal increase in risk stratification (NRI 0.52 [95% CI 0.40, 0.64], *p* < 0.05; IDI 0.03 [95% CI 0.02, 0.04]; *p* > 0.05)

A metabolite score was derived from the five most robustly predictive metabolites, i.e. DAG(16:0/18:1), lysoPC(19:1), PC(17:0/18:2), isoleucine and l-tyrosine (Fig. 3a). Adding this score to model 1 or model 2 significantly improved prediction, e.g. AUC_ROC_ increased by around 4% (*p* < 0.001). The optimal TS consisted of 2 h-PG, FPG, BMI, total cholesterol, triacylglycerols, systolic BP and red and processed meat intake among cohort-specific traditional risk factors, and showed significantly improved prediction compared with model 1, model 2 and metabolite score (AUC_ROC_ increased by 5.4%, 8.3% and 6.8%, respectively, *p* < 0.001). The addition of metabolite score to TS marginally increased AUC_ROC_ by 2.6% (Fig. 3b). Interestingly, when a metabolite score was applied to the participants who were misclassified by the TS, 36 of the 80 misclassified participants at high risk (45%) were correctly classified as later cases. On excluding 2 h-PG from TS (i.e. TS-_2h-PG_), this risk score still showed better prediction compared with model 1 or 2, and addition of robust predictive metabolites to TS-_2h-PG_ significantly increased AUC_ROC_ by 2.7% (ESM Fig. 5). The optimal selection of variables for the CS included nine predictors: FPG, 2 h-PG, triacylglycerols, BMI, lysoPC(19:1), lysoPC(18:2), DAG(14:0/18:1), *N*-acetylglycine and isoleucine, and had comparable AUC_ROC_ to the TS.

Most metabolites were positively or negatively correlated with BMI and 2 h-PG (*r* = −0.4~0.5, *p <* 0.0001; ESM Fig. 6). DAGs were strongly correlated with triacylglycerols (*r* = 0.45~0.64, *p <* 10^−9^). Moreover, 41 of the metabolites were directly correlated with HOMA-IR and/or HOMA-%B at baseline (Table 2, ESM Fig. 7). The correlations between some metabolites, e.g. BCAAs, lysoPCs, DAGs and HOMA-%B, were attenuated at the 10 year follow-up.

## Discussion

In this large nested case–control study, we explored predictive metabolites of type 2 diabetes by adopting untargeted metabolomics in combination with a robust data processing pipeline. By using the repeated samples collected 10 years after baseline, we found that the long-term reproducibility in healthy controls was modest to excellent for a majority of the metabolites, strengthening their potential as predictive biomarkers in clinical studies; several metabolites had changed in the disease-associated direction at follow-up, potentially representing disease progression. Importantly, our comprehensive prediction analyses illustrated that single measurements of predictive metabolites can provide complementary but limited information beyond the optimal use of traditional risk factors in relation to risk prediction.

### Predictive metabolites of type 2 diabetes

We discovered for the first time that lysoPC(19:1) and PC(17:0/18:2) were associated with higher insulin sensitivity and inversely associated with type 2 diabetes. Two previous studies have reported an inverse association between PCs containing odd-chain fatty acids and type 2 diabetes [10, 30], but none have assessed reproducibility. Herein, we found high long-term reproducibility (ICC ≥0.4) of lysoPC(19:1) and PC(15:1/18:2) among healthy controls, reinforcing their potential as predictive biomarkers. Plasma odd-chain fatty acids are often considered as markers of dairy product intake but can also be formed endogenously in adipocytes through α-oxidation of palmitic and stearic acid [31, 32] or other biosynthesis [32]. We found no correlation of these PCs with milk, yogurt or cheese. More studies are needed to improve our understanding of PCs containing odd-chain fatty acids in type 2 diabetes and for mechanistic investigations.

We explored 2-hydroxyethanesulfonate as a novel predictive metabolite with moderate reproducibility (ICC 0.34 [95% CI 0.23, 0.47]). 2-Hydroxyethanesulfonate is a downstream metabolite of taurine, possibly formed by anaerobic gut bacteria [33] or myeloperoxidase-induced degradation. It correlated with triacylglycerols (*r* = −0.2, *p <* 0.001) and increased with antihypertensive and/or lipid-controlling medication, suggesting a role in lipid metabolism. The lower level of 2-hydroxyethanesulfonate among cases supports a proposed link between taurine metabolism disturbance and diabetes [34, 35].

We also confirmed several metabolites that have previously been reported. Replicating previous findings in an external study with a larger number of cases brings strong supportive evidence to previous findings. The observed strong associations of these metabolites with future type 2 diabetes support existing hypotheses in relation to the pathogenesis of type 2 diabetes, e.g. dysregulated lipid metabolism (PCs) [36, 37], impaired BCAA metabolism (BCAAs and their metabolites) [38, 39], abnormal DAG accumulation that interferes with cellular signalling (DAGs) [37], and the role of the small intestine in the control of glucose homeostasis (citrulline) [40]. We also replicated the inverse association for *N*-acetylglycine [6], but the mechanisms remain unknown. *N*-Acetylglycine has been related to gut bacterial metabolism and positively correlated with dietary fibre intake [41], but this could not be confirmed at present (*r* < 0.1).

Importantly, our study provides information of reproducibility of previously identified predictive metabolites, which has rarely been investigated before. Specifically, four out of seven PCs, i.e. lysoPC(18:2), lysoPC(17:0), lysoPC(20:1) and PC(16:0/16:1), had weak to moderate long-term reproducibility (ICC <0.4) in the present study, which limits their use as predictive biomarkers. BCAAs and 3-methyl-2-oxovaleric acid had high long-term (10 years) reproducibility (ICC >0.6), similar to the 2 year ICCs [42] but weaker than the shorter term reproducibility [43, 44].

### Changes in metabolites over time among type 2 diabetes cases

We found that BCAAs, 3-methyl-2-oxovaleric acid and glutamate increased at follow-up among cases, while PCs containing odd-chain fatty acids decreased, regardless of time to diagnosis or use of medication. By contrast, medication appeared to affect changes in some metabolites at follow-up, such as DAGs, phenylalanine, tryptophan, *N*-acetylglycine and 2-hydroxyethanesulfonate (ESM Table 7). This may explain the attenuated correlations between metabolites and HOMA-%B at follow-up (ESM Fig. 7). Metabolites changed over time along with disease progression, and their responses to medication may provide novel candidate targets for therapeutic intervention, as well as biomarkers of both disease progression and treatment efficacy. These preliminary findings merit further investigation using appropriate study settings, e.g. randomised controlled trials [45].

### Improved type 2 diabetes risk prediction with optimal variable selection

Our results showed that optimal use of a single measurement of metabolites at baseline could improve risk prediction when combined with traditional risk factors that are typically measured in observational studies [4, 8, 15–18]. However, optimal selection among traditional risk factors is probably of even greater importance (Fig. 3).

Similar to other studies, adding selected predictive metabolites to models constructed using predefined risk factors that have been used as covariates adjusted in prediction models improved risk prediction by 3–5%. The optimal study-specific selection of risk factors (TS approach) led to a larger improvement in risk prediction (6–8%) compared with commonly used predefined risk predictors. Of note, when using the optimal selected traditional risk factors (TS or TS-_2h-PG_) as reference, metabolites still improved the prediction somewhat, but not to the same extent as when added to predefined risk factor models (models 1 and 2). Therefore, our results suggest that the substantial improvements with the addition of potential metabolite biomarkers observed and highlighted in previous studies may be due to comparison with suboptimally selected traditional risk predictors and may not represent true improvements, due to complementarity of metabolite information. To our knowledge, this has not been discussed in previous studies and needs to be confirmed in other populations.

Many disease risk predictors typically used in cohort studies, e.g. single measurements of blood lipids, BP, and dietary data from a food frequency questionnaire, suffer from large systematic and random errors, which in turn may lead to inaccurate risk estimates [46]. Although providing little additional benefit in predictive performance to the TS, we observed that the selected metabolite predictors in CS showed higher long-term reproducibility (0.37 ≤ ICC ≤ 0.68) compared with total cholesterol, systolic BP and consumption of red and processed meat (0.3 ≤ ICC ≤ 0.41) involved in TS, suggesting that optimal use of reproducible metabolites may improve statistical power, provide more accurate risk estimates and under some conditions serve as a complement or alternative to established risk factors.

### Strengths and limitations

Our study has some limitations. First, as an observational study it cannot confirm causality of the findings. Second, although we applied robust internal validation for evaluation of predictive metabolites, external validation of findings in independent cohorts is still warranted. Despite this limitation, 20 out of 30 metabolites (MSI 1–2) highlighted in this study as top-ranked predictors had previously been reported, suggesting that our results are robust. Third, for risk prediction, due to the lack of available measurements of LDL- and HDL-cholesterol and the missing data on waist circumference, we could not calculate established risk scores, such as the Diabetes Risk Score or the Framingham Risk Score, as references to compare with identified predictive metabolites. Moreover, multicollinearity is often a problem in epidemiology when constructing prediction models. Herein, we are aware of the inherent multicollinearity between covariates used in model 3, i.e. BP, total cholesterol and triacylglycerols, but this multicollinearity did not change the overall interpretation for the associations between metabolites and risk of type 2 diabetes. Finally, even with extensive efforts on metabolite identification, we did not manage to annotate all discriminative features. Unknown metabolites (MSI 3–4) might theoretically have provided additional predictive power if included in the models. However, the identified five best predictive metabolites were consistently selected from the dataset among identified metabolites (MSI 1–2), with or without unknown metabolites, suggesting limited additional benefits of adding yet unidentified metabolites to the models. Importantly, we focused on identified metabolites to arrive at results that can be interpreted, potentially reproduced and translated across studies.

Our study also has several strengths. First, it constitutes the hitherto largest untargeted metabolite profiling study of incident type 2 diabetes in a nested case–control setting. Second, the availability of repeated samples 10 years after baseline for a subset allowed us to investigate both long-term reproducibility of metabolites and changes in relation to disease diagnosis and medication. Third, for the first time, we comprehensively compared risk predictability between different risk models including metabolites as well as traditional risk factors a priori selected or optimally selected, highlighting the greater potential of optimal use of traditional risk factors.

In conclusion, we explored novel and previously identified predictive metabolites of type 2 diabetes, providing complementary and additional information to improve understanding of the pathophysiology of type 2 diabetes. However, single measurements of predictive metabolites can only provide limited information beyond the optimal use of traditional risk factors in relation to risk prediction.

## Electronic supplementary material

ESM (PDF 1.91 mb)

## Abbreviations

2 h-PG: 2 h plasma glucose
AUC_ROC_: Area under the receiver operating characteristic curve
BCAA: Branched-chain amino acid
CS: Combined score
DAG: Diglyceride
FPG: Fasting plasma glucose
HOMA-%B: HOMA-derived beta cell function
ICC: Intra-class correlation
LC: Liquid chromatography
ML-PLS: Multilevel partial least squares
MSI: Metabolomics Standard Initiative
PC: Phosphatidylcholine
ROC: Receiver operating characteristic curve
RT: Retention time
TS: Traditional risk score

## References

[1] Stumvoll M, Goldstein BJ, van Haeften TW (2010). Type 2 diabetes: principles of pathogenesis and therapy. Lancet.

[2] Klein MS, Shearer J (2016). Metabolomics and type 2 diabetes: translating basic research into clinical application. J Diabetes Res.

[3] Dunn WB (2012). Diabetes—the role of metabolomics in the discovery of new mechanisms and novel biomarkers. Curr Cardiovasc Risk Rep.

[4] Herder C, Kowall B, Tabak AG, Rathmann W (2014). The potential of novel biomarkers to improve risk prediction of type 2 diabetes. Diabetologia.

[5] Sas KM, Karnovsky A, Michailidis G, Pennathur S (2015). Metabolomics and diabetes: analytical and computational approaches. Diabetes.

[6] Menni C, Fauman E, Erte I (2013). Biomarkers for type 2 diabetes and impaired fasting glucose using a nontargeted metabolomics approach. Diabetes.

[7] Drogan D, Dunn WB, Lin W (2014). Untargeted metabolic profiling identifies altered serum metabolites of type 2 diabetes mellitus in a prospective, nested case–control study. Clin Chem.

[8] Zhao J, Zhu Y, Hyun N (2015). Novel metabolic markers for the risk of diabetes development in American Indians. Diabetes Care.

[9] Fall T, Salihovic S, Brandmaier S (2016). Non-targeted metabolomics combined with genetic analyses identifies bile acid synthesis and phospholipid metabolism as being associated with incident type 2 diabetes. Diabetologia.

[10] de Mello VD, Paananen J, Lindström J (2017). Indolepropionic acid and novel lipid metabolites are associated with a lower risk of type 2 diabetes in the Finnish Diabetes Prevention Study. Sci Rep.

[11] Qiu G, Zheng Y, Wang H (2016). Plasma metabolomics identified novel metabolites associated with risk of type 2 diabetes in two prospective cohorts of Chinese adults. Int J Epidemiol.

[12] Abbasi A, Stolk RP, Bakker SJ (2014). Identification of relevant biomarkers for type 2 diabetes. Lancet Diabetes Endocrinol.

[13] Kotsopoulos J, Tworoger SS, Campos H (2010). Reproducibility of plasma, red blood cell, and urine biomarkers among premenopausal and postmenopausal women from the Nurses’ Health Studies. Cancer Epidemiol Biomark Prev.

[14] Spiegelman D, McDermott A, Rosner B (1997). Regression calibration method for correcting measurement-error bias in nutritional epidemiology. Public Health.

[15] Lu Y, Wang Y, Ong CN (2016). Metabolic signatures and risk of type 2 diabetes in a Chinese population: an untargeted metabolomics study using both LC-MS and GC-MS. Diabetologia.

[16] Peddinti G, Cobb J, Yengo L (2017). Early metabolic markers identify potential targets for the prevention of type 2 diabetes. Diabetologia.

[17] Wang TJ, Larson MG, Vasan RS (2011). Metabolite profiles and the risk of developing diabetes. Nat Med.

[18] Yengo L, Arredouani A, Marre M (2016). Impact of statistical models on the prediction of type 2 diabetes using non-targeted metabolomics profiling. Mol Metab.

[19] Norberg M, Wall S, Boman K, Weinehall L (2010). The Västerbotten Intervention Programme: background, design and implications. Glob Health Action.

[20] Rolandsson O, Norberg M, Nyström L (2012). How to diagnose and classify diabetes in primary health care: lessons learned from the Diabetes Register in Northern Sweden (DiabNorth). Scand J Prim Health Care.

[21] Brunius C, Shi L, Landberg R (2016). Large-scale untargeted LC-MS metabolomics data correction using between-batch feature alignment and cluster-based within-batch signal intensity drift correction. Metabolomics.

[22] Brown M, Wedge DC, Goodacre R (2011). Automated workflows for accurate mass-based putative metabolite identification in LC/MS-derived metabolomic datasets. Bioinformatics.

[23] van Velzen EJJ, Westerhuis JA, van Duynhoven JPM (2008). Multilevel data analysis of a crossover designed human nutritional intervention study. J Proteome Res.

[24] Buck M, Nilsson LKJ, Brunius C, Dabiré RK, Hopkins R, Terenius O (2016). Bacterial associations reveal spatial population dynamics in *Anopheles gambiae* mosquitoes. Sci Rep.

[25] Hanhineva K, Brunius C, Andersson A (2015). Discovery of urinary biomarkers of whole grain rye intake in free-living subjects using nontargeted LC-MS metabolite profiling. Mol Nutr Food Res.

[26] Therneau TM, Grambsch PM (2000). Modeling survival data: extending the Cox model.

[27] Robin X, Turck N, Hainard A (2011). pROC: an open-source package for R and S+ to analyze and compare ROC curves. BMC Bioinform.

[28] Kundu S, Aulchenko YS, van Duijn CM, Janssens ACJW (2011). PredictABEL: an R package for the assessment of risk prediction models. Eur J Epidemiol.

[29] Sumner LW, Amberg A, Barrett D (2007). Proposed minimum reporting standards for chemical analysis. Metabolomics.

[30] Meikle PJ, Wong G, Barlow CK (2013). Plasma lipid profiling shows similar associations with prediabetes and type 2 diabetes. PLoS One.

[31] Jenkins B, West JA, Koulman A (2015). A review of odd-chain fatty acid metabolism and the role of pentadecanoic acid (C15:0) and heptadecanoic acid (C17:0) in health and disease. Molecules.

[32] Jenkins BJ, Seyssel K, Chiu S (2017). Odd chain fatty acids; new insights of the relationship between the gut microbiota, dietary intake, biosynthesis and glucose intolerance. Sci Rep.

[33] Zdenek K, Hollemeyer K, Smits THM, Cook AM (2010). Isethionate formation from taurine in *Chromohalobacter salexigens*: purification of sulfoacetaldehyde reductase. Microbiology.

[34] Ito T, Schaffer SW, Azuma J (2012). The potential usefulness of taurine on diabetes mellitus and its complications. Amino Acids.

[35] Zheng Y, Ceglarek U, Huang T (2016). Plasma taurine, diabetes genetic predisposition, and changes of insulin sensitivity in response to weight-loss diets. J Clin Endocrinol Metab.

[36] Meikle PJ, Summers SA (2017). Sphingolipids and phospholipids in insulin resistance and related metabolic disorders. Nat Rev Endocrinol.

[37] Markgraf D, Al-Hasani H, Lehr S (2016). Lipidomics—reshaping the analysis and perception of type 2 diabetes. Int J Mol Sci.

[38] Chen T, Ni Y, Ma X (2016). Branched-chain and aromatic amino acid profiles and diabetes risk in Chinese populations. Sci Rep.

[39] Lotta LA, Scott RA, Sharp SJ (2016). Genetic predisposition to an impaired metabolism of the branched-chain amino acids and risk of type 2 diabetes: a Mendelian randomisation analysis. PLoS Med.

[40] Verdam FJ, Greve JWM, Roosta S (2011). Small intestinal alterations in severely obese hyperglycemic subjects. J Clin Endocrinol Metab.

[41] Lustgarten MS, Price LL, Chale A, Fielding RA (2014). Metabolites related to gut bacterial metabolism, peroxisome proliferator-activated receptor-alpha activation, and insulin sensitivity are associated with physical function in functionally-limited older adults. Aging Cell.

[42] Carayol M, Licaj I, Achaintre D (2015). Reliability of serum metabolites over a two-year period: a targeted metabolomic approach in fasting and non-fasting samples from EPIC. PLoS One.

[43] Floegel A, Drogan D, Wang-Sattler R (2011). Reliability of serum metabolite concentrations over a 4-month period using a targeted metabolomic approach. PLoS One.

[44] Breier M, Wahl S, Prehn C (2014). Targeted metabolomics identifies reliable and stable metabolites in human serum and plasma samples. PLoS One.

[45] Rankin NJ, Preiss D, Welsh P, Sattar N (2016). Applying metabolomics to cardiometabolic intervention studies and trials: past experiences and a roadmap for the future. Int J Epidemiol.

[46] Tirosh A, Shai I, Bitzur R (2008). Changes in triglyceride levels over time and risk of type 2 diabetes in young men. Diabetes Care.

[47] Peters T, Brage S, Westgate K (2012). Validity of a short questionnaire to assess physical activity in 10 European countries. Eur J Epidemiol.

[48] Floegel A, Stefan N, Yu Z (2013). Identification of serum metabolites associated with risk of type 2 diabetes using a targeted metabolomic approach. Diabetes.

[49] Ferrannini E, Natali A, Camastra S (2013). Early metabolic markers of the development of dysglycemia and type 2 diabetes and their physiological significance. Diabetes.

