# Supplementary material for: Plasma metabolites associated with type 2 diabetes in a Swedish population: a case–control study nested in a prospective cohort
Source: Diabetologia. 2018 Jan 18;61(4):849–61. doi: 10.1007/s00125-017-4521-y (PMC6448991; doi:10.1007/s00125-017-4521-y)
Supplement: Supplementary file 1 — (PDF 1.91 mb) [file 125_2017_4521_MOESM1_ESM.pdf]

Electronic Supplementary Material (ESM)

Plasma metabolites associated with type 2 diabetes in a Swedish population: a case-control study nested in a prospective cohort

ESM Methods

Västerbotten Intervention Programme cohort

The Västerbotten Intervention Programme (VIP) is one of the sub-cohorts of the Northern Sweden Health and Disease Study cohort [1]. The cohort included 141,000 sampling occasions from 98,300 unique individuals by March 2015, out of which 36,100 individuals have provided repeated samples. Recruitment for the VIP cohort started in 1985. Individuals from Västerbotten County at ages 30, 40, 50 and 60 years were invited to participate in systematic risk factor screening program including individual counselling about lifestyle habits, at their local health care center. An oral glucose tolerance test following WHO standards was conducted with a 75-g oral glucose load among all except for participants with previously known diabetes mellitus or with fasting glucose exceeding the criterion for diabetes (≥7.0 mmol/l). Blood samples were drawn and stored at the Northern Sweden Medical Biobank. All participants were asked to complete a questionnaire at all assessments, which captured information on socioeconomic conditions, self-rated health, personal health history and family history of diabetes, tobacco use and physical activity [1]. Two modified and validated versions of the Northern Sweden food frequency questionnaires were used: one with 84 food items and another with 64 items [2,3].

Analytical protocol of Untargeted LC-MS metabolomics

Fasting heparin plasma samples (90 µl) was mixed with 360 µl acetonitrile (VWR International), incubated in a 96 deep well plate (RNase/DNase-Free 1 ml well, Thermo) on an

ice bath for 15 min and then centrifuged at 1200 g for 5 min (Megafuge 40R centrifuge, Thermo) through a 0.2 µm polytetrafluoroethylene filters to collect the filtrate. The filtrate were kept in refrigerator at 4℃ until they were injected. Two types of independent biological samples (QC A: batch-specific quality control sample and QC B: a long-term reference sample) were used to monitor the stability and functionality of the system throughout the instrumental analyses and together constituted approximately 16 % of analytical samples, as described by Brunius et al. 2016 [4]. The samples were analyzed in 8 batches with randomisation being constrained to having sample pairs and repeated samples within the same batch, and otherwise full randomisation within batch. Instrumental analyses were performed with approximately 250 injections per batch in 8 analytical batches over half a year.

Sample solution (4 µl) from protein-precipitated heparin plasma, described in the main text, was injected for reversed-phase (RP) chromatographic analyses. Separation was performed using a Zorbax Eclipse XDB-C18 column (2.1 x 100 mm, 1.8 mm; Agilent Technologies) at 50℃. The mobile phase was delivered at 400 µL /min and consisted of eluent A (water, Milli-Q purified; Millipore) and eluent B (methanol, Sigma-Aldrich), both containing 0.1% (vol:vol) of formic acid (Sigma-Aldrich), delivered in a gradient profile: 0-10 min 2 to 100% B, 10-14.5 min: 100% B, 14.5- 14.51 min: 100 to 2% B, 14.51- 16.5: 2% B.

Sample (3 µl) was injected for hydrophilic interaction chromatography (HILIC) analyses. An Acquity UPLC BEH Amide column (2.1 x 100 mm, 1.7 mm; Waters Corporation) was used and maintained at 45℃ for separation. The mobile phase was delivered at 600 µL/min and consisted of 50% acetonitrile (vol:vol; eluent A) and 90% acetonitrile (vol:vol; eluent B), respectively, both containing 20 mmol/l ammonium formate (pH 3 ,Sigma-Aldrich), delivered in a gradient profile: 0-2.5 min: 100% B, 2.5-10 min: 100 to 0%B, 10-10.01 min: 0 to 100% B, 10.01- 12.5 min: 100% B.

The ESI source was operated using the following conditions: Drying gas (nitrogen) temperature of 325℃ and flow of 10 L/min, sheath gas temperature of 350℃ and flow of 11 L/min, nebulizer pressure of 45 PSI, capillary voltage of 3500 V, nozzle voltage of 1000 V, fragmentor voltage of 100 V, and a skimmer of 45V. For data acquisition, a 2-GHz extended dynamic range mode was used, and the instrument was set to acquire over the mass range of m/z 20–1600. Data were collected in centroid mode at an acquisition rate of 1.67 spectra/s with an abundance threshold of 150 counts. The approach for automatic data-dependent MS/MS analyses was described elsewhere [5]. Importantly, collision energies were 10, 20, 40 V in subsequent runs. In addition, targeted MS/MS analyses with collision energies 10 and 20 V

were conducted for the molecular ions not included into automatic data-dependent MS/MS fragmentation. Continuous mass axis calibration was performed by monitoring two reference ions, m/z 121.050873 and m/z 922.009798 for positive mode and m/z 113.988900 and 966.000725 for negative mode, from an infusion solution throughout the runs.

Data pre-processing

Raw data files from HILIC (ESI+), HILIC (ESI-), RP (ESI+), RP (ESI-) were converted to mzXML format using MassHunter Qualitative Analysis B.06.00 (Agilent Technologies). Peak picking was performed with XCMS, a freely available software under open-source license, implemented in R. Functions of all parameters involved in XCMS have been described elsewhere[6]. Since parameter settings in XCMS have large impact on the number and quality of the identified features and they are instrument-dependent and study-specific [7,8], optimization of main parameters was conducted for each mode separately, as described below.

Peak detection, retention time correction and peak alignment

We performed peak detection in each chromatogram using the *centWave* algorithm implemented in the *xcmsSet* function. Parameters, such as *ppm*, *snthresh*, and *peakwidth* are important to capture well-behaved features and they are strongly instrument-specific[9]. Iterative testing of different settings of parameter configurations was used to identify the potential range of parameters setting. The initial interval of parameters were the values suggested by XCMS online (https://xcmsonline.scripps.edu/) and recently relevant publications [8,10,11]. IPO, an R package for automated optimization of XCMS parameters was then applied to determine optimal parameter values [12]. The quality of algorithm performance was evaluated by the number of detected features and visualizing plot of detected peaks, with the *findFeature* function. Retention time correction was achieved using the *Obiwarp* function for HILIC (ESI+), HILIC (ESI-), and RP (ESI-). For RP (ESI+), the *Obiwarp* method produced errors and the *LOESS* fitting method was used instead [6].

Correction of feature misaligned between batches

The R-based pipeline, ‘batchCorr’ [4] was used for alignment and merging of features that were systematically misaligned between batches. This procedure aggregates present/missing features on batch level and combines similar features orthogonally present between batches. Importantly, *rtdiff* is equal to retention time deviation of samples in 8 batches. The *rtdiff* parameter setting for HILIC (ESI+), HILIC (ESI-), RP (ESI+), RP (ESI-), were 10, 15, 10, 15 seconds, respectively. We applied *mzdiff* 0.005 for the 4 different modes. For the reason that

QC A were of the same biological origin as the real samples, and therefore representative of sample population, batch alignment was performed depending on QC A information.

Within- and between- batch feature intensity drift correction

Within- and between-batch measurement errors, due to shifts in retention time, mass-to-charge ratio (m/z) and intensity of features between analytical runs, severely affect subsequent statistical analysis and further metabolite identification. A cluster-based within-batch intensity drift correction was performed batch-wise [4]. Normalization was applied on features detected in at least 60% of the QCAs per batch. Features passing a QCA test (CV <0.3) were determined as batch-specific qualified features. Features were then over-all retained if they were qualified in at least 5 batches. Between-batch normalization was then applied on those collected features using a limit value for fold change (FC) between batches to determine the feature-wise choice of reference-based or population-based between-batch normalization. FC limits were 7,5,5,5 for RP (ESI+), RP (ESI-), HILIC (ESI+), and HILIC (ESI-), respectively.

PUTMEDID-LCMS Workflows

Putative annotation of metabolites or metabolite groups was performed by applying the PUTMEDID-LCMS Workflow operating in the Taverna environment [13]. The peak table after batch misalignment correction was used for ion annotation following the three workflows as described in operating instructions of PUTMEDID-LCMS Workflows (http://www.mcisb.org/resources/putmedid.html). We applied 10 ppm mass error and a retention time range of 2.5s in feature grouping and molecular formula. The annotation was performed batch-wise for each mode. Features with a retention time <30 or > 720 s in HILIC chromatography or with a retention time <70 or > 980 s in RP chromatography were excluded from annotation. For each feature, qualified annotation was determined only if same annotation was given at least in five batches. In total, 29,240 features were considered qualified after a stringent normalization procedure.

We also manually checked and removed features with RT 4.88 ± 0.63 min and features with RT <1.16 min from HILIC data to remove the over-representation of hexose features and poorly retained lipids in HILIC.

ESM Tables

ESM Table 1. Baseline characteristics of the total participants (503 pairs), participants with (187 pairs) and without (316 pairs) repeated samples in the Västerbotten Intervention Programme cohort.

Characteristics	Cases			Matched controls		
	All (n=503)	No repeated samples (n=316)	Have repeated samples(n=187)	All (n=503)	No repeated samples(n=316)	Have repeated samples(n=187)
Men[a]	44.5	38.9	54	44.5	38.9	54
Age, year[a]	50.2 (7.9)	52.6(7.9)	45.9(5.9)	50.1 (8.0)	52.6(7.9)	45.9(5.9)
Fasting glucose, mmol/l)	6.0 (0.9)	6.1(0.9)	5.9(0.9)	5.5 (1.1)	5.4(0.8)	5.5(1.4)
2 h-plasma glucose, mmol/l)	8.3 (2.8)	8.5(2.7)	7.8(2.6)	6.5 (1.6)	6.6(1.7)	6.3(1.3)
BMI, kg/m^2	29.5 (4.9)	29.8(5.1)	28.9(4.5)	25.5 (3.8)	25.6(4.14)	25.3(3.2)
HOMA-IR	NA	NA	1.7(1.2)	NA	NA	0.9(0.7)
HOMA-B%[b]	NA	NA	101.5(72.9)	NA	NA	74.5(27.7)
Triacylglycerols, mmol/l	2.0 (1.3)	2.1(1.2)	1.8(1.2)	1.4 (0.7)	1.4(0.7)	1.3(0.6)
Total cholesterol, mmol/l	5.9 (1.2)	6(1.2)	5.7(1.2)	5.7 (1.1)	5.8(1.1)	5.5(1.1)
Systolic BP, mmHg	138 (18.1)	140(18.5)	135.4(16.8)	128 (17.2)	129(17.5)	126.4(16.4)
Diastolic BP, mmHg	85 (10.4)	84.7(10.4)	84.7(10.4)	80 (9.7)	79.5(9.8)	76.6(10.2)
Total energy intake, kJ/day	7184.4(2555.9)	7016.3 (2485.8)	7454.5 (2643.4)	7282.4 (2590.8)	7072.4(2461.3)	7632.5(2783.4)
Dietary fibre, g/day	18.9 (7.4)	18.9(7.5)	18.9(7.4)	19.5(8.2)	19.6(7.8)	19.4(8.9)
Whole grain, g/day	72.2(36.5)	71.9 (28.2)	72.9(36.4)	74.4(39.6)	75.1(29.8)	73.7(39.5)

Fat, g/day	68.2 (26.0)	62.3(27.7)	70.1(30.5)	64.3(27.7)	61.3(25.7)	69.3(30.3)
Alcohol, g/day	3.3(6.7)	3.6(8.1)	3(2.8)	3.6(4.3)	3.4(4.3)	4.06(4.32)
Smoking status						
Current smoker	22.4	21	24.5	19.1	20.9	15.5
Former smoker	28.6	30	26.7	25.8	24.3	29.4
Occasional smoker	1.0	1.6	0	3.8	2.5	5.8
Former occasional smoker	9.2	7.3	12.3	7.2	6.3	8.5
Non-smoker	38.7	40.5	35.8	43.9	45.9	39.5
Physical activity						
Inactive	18.7	19.9	16.1	17.9	15.8	21.3
Moderately inactive	35.4	36.4	34.2	35.9	36.1	35.8
Moderately active	28.2	28.2	28.3	27.3	28.1	25.6
Active	17.7	15.5	20.8	18.9	19.9	17.5
Education						
Elementary school	33.3	36.4	27.8	29.8	30.7	27.8
Vocational (training) school	28.1	27.2	29.9	26.2	26.6	26.2
Secondary school	22.3	20.5	25.6	20.6	19.9	21.9
University education/college	16.3	16.1	16.5	23.4	22.8	24.1

Data are mean (SD) or %.

[a]Matching factors.

[b]The percent of HOMA-beta cell function.

ESM Table 2. Characteristics of the subset of 187 pairs of cases and control at baseline and after 10 years follow up in the Västerbotten Intervention Programme cohort.

Characteristics	Group[a]	Cases(n=187)		Matched controls(n=187)	
		Baseline	Follow up	Baseline	Follow up
Number	A	26	26	26	26
	B	52	52	52	52
	C	109	109	109	109
Men (%)[b]	A	46	46	46	46
	B	62	62	62	62
	C	52	52	52	52
BMI, kg/m^2 [c]	A	27.5(3.7)	29.9(4.4)	24.7(3.1)	26.59(3.7)
	B	27.4(4.0)	29.3(4.7)	24.82(3.7)	25.71(3.8)
	C	30.0(4.6)	30.7(4.9)	25.72(2.9)	26.62(3.7)
Age, years[b,c]	A	45.8(5.8)	55.8(5.6)	45.9(5.8)	55.9(5.8)
	B	43.6(5.8)	53.6(5.8)	43.6(5.9)	53.6(5.9)
	C	47.0(5.6)	57.0(5.5)	47.1(5.6)	57.1(5.5)
Fasting plamsa glucose, mmol/l[c]	A	5.7(0.8)	7.2(2.3)	5.2 (0.4)	5.65(0.5)
	B	5.8(0.7)	8.6(2.7)	5.3(0.5)	5.54(0.7)
	C	6.1(0.8)	8.3(2.2)	5.7(1.8)	5.65(0.9)
2 h-plasma glucose, mmol/l[d]	A	7.9(2.7)	NA	5.77(1.3)	6.84(2.2)
	B	7.5(1.9)	NA	6.11(1.3)	6.69(1.9)
	C	7.9(2.9)	NA	6.62(1.3)	6.95(1.9)
Triacylglycerols, mmol/l[e]	A	1.8(0.9)	2.2(1.2)	1.02(0.3)	1.34(0.5)
	B	2.0(1.5)	1.97(1.2)	1.26(0.5	1.39(0.7)
	C	1.8(1.9)	1.61(0.7)	1.37(0.7	1.44(0.7)
Total cholesterol, mmol/l	A	5.6(1.2)	5.68(1.0)	5.37(0.9)	5.3(1.5)
	B	5.6(1.1)	5.29(1.4)	5.61(1.3)	5.45(0.9)
	C	5.7(1.4)	4.79(0.9)	5.49(1.1)	5.49(1.1)
Systolic BP, mmHg	A	137.3(22.2)	145.74(20.4)	131(16.1)	130(16.6)
	B	133.2(17.6)	139.57(19.4)	122(14.5)	130.8(18.9)
	C	136.1(15.1)	134.19(16.3)	127.6(17.1)	131.8(16.1)
Diastolic BP, mmHg	A	84.5(13.0)	82.2(9.0)	83.4(9.0)	78.8(10.9)
	B	84.6(10.8)	84.4(10.2)	77.9(10.7)	80.0(11.7)
	C	84.9(9.6)	82.3(9.1)	79.5(10.0)	80.9(10.3)

Data are mean (SD).

[a]187 pairs of participants were assigned into three groups, depending on when the diagnosis was made in relation to the second sample: group A, where the second sample was drawn

before (median 2 years) diagnosis (n=26 pairs); group B, where the second sample was drawn in the same year as diagnosis (n=52 pairs); and group C, where the second sample was drawn after (median 4 years) diagnosis (n=109 pairs).
[b]Matching factors.
[c]Paricipants in group C tended to have higher age, BMI, and fasting plasma glucose (unadjusted p<0.05).
[d]Among 187 cases, 109 were lacking 2h-plasma glucose at 10 year of follow up. The mean value is likely not representative for the subset population and is therefore not provided.
[e]Triacylglycerols was lower at follow-up compared to baseline in subset of cases whose follow-up sample was drawn in the same year as diagnosis or after diagnosis. Such reduction may be attributed to the medication.

ESM Table 3. Classification of prescribed drugs among the subset of 187 type 2 diabetes cases with follow-up samples 10 years after baseline.

Type	Medication	Number of participants
Glucose-lowering	Insulin or Insulin analog	30
Glucose-lowering	Metformin	105
Glucose-lowering	Sulfonylureas	14
Glucose-lowering	Alpha-glucosidase inhibitors	0
Glucose-lowering	Thiazolidinediones	2
Glucose-lowering	Other oral antidiabetics and past oral treatment	6
Others	Antihypertensive medication	127
Others	Lipid lowering medication	117
Others	Corticosteroid therapy	8

ESM Table 4. Optimized parameters applied for untargeted LC-MS data processing.

Parameters	Chromatography and ionisation mode[a]			
	HILIC (ESI+)	HILIC (ESI-)	RP(ESI+)	RP (ESI-)
XCMS[b]				
snthresh	6	6	6	6
ppm	15	15	15	15
prefilter	(3,1000)	(3,1000)	(3,1000)	(3,1000)
Peakwidth	(5,20)	(5,20)	(5,76)	(5,84)
mzdiff	0.0056	0.005	0.0045	0.0045
gapInit	0.928	0.296		0.58
gapExtend	2.688	2.4		2.19
bw	1.5	1.5	30, 10, 5 *	2
batchCorr[c]				
mzdiff	0.005	0.005	0.005	0.005
rtdiff	10	15	10	15
Fold change limit	5	5	7	5
PUTMEDID-LCMS[d]				
ppm mass error	10	10	10	10
Retention time range	2.5	2.5	2.5	2.5

[a] RP: Reverse phase chromatography; HILIC: hydrophilic interaction chromatography. '+' or '–' denotes the electrospray ionization, positive or negative, respectively.

[b]A freely available software implemented in R was applied for data deconvolution. See further details in ESM Methods. Retention time correction was achieved using the *Obiwarp* function for HILIC (ESI+), HILIC (ESI-), and RP (ESI-) and *LOESS* fitting method was used for RP (ESI+). snthresh: signal to noise ratio; ppm: measurement error; prefilter: prefiltering step for the first analysis step (ROI detection), mass traces are only retained if they contain at least 3 peaks with intensity >= 1000; Peakwidth: the expected approximate peak width in chromatographic space, given as a range (min, max) in seconds; mzdiff: the minimum difference in m/z dimension for peaks with overlapping retention times; gapInit and gapExtend : penalty for gap opening and gap enlargement, respectively; bw: the bandwidth (standard deviation of the smoothing kernel) for alignment.*Peak group retention time correction and alignment were performed with different bandwidth setting iteratively.

[c]The R-based pipeline, 'batchCorr' was used for within- and between-batch correction. *rtdiff* is equal to retention time deviation of samples in 8 batches. See further details in ESM Methods.

[d]A freely available workflow for putative annotation of metabolites or metabolite groups (http://omictools.com/putmedid-lcms-tool). See further details in ESM Methods.

ESM Table 5. The characteristics and identification of 46 predictive metabolites of type 2 diabetes.

Chromatography[a]	Ionisation Mode[b]	Metabolites	Mass	RT (min)	MSI[c]	**Database ID**	MS/MS fragmentation
HILIC	+	2-Methylbutyroylcarnitine	245.1626	1.28	2	MID 5367[d]	ESI+ 246.1099, 187.0907, 85.0295
HILIC	+	161.0062@2.7	161.0062	2.70	4		ESI+ 162.0134, 121.049,54.203
HILIC	+	3-Hydroxyisovalerylcarnitine	261.1575	3.20	2	MID 6505 [d]	ESI+ 262.1648, 185.082, 145.048, 41.192
HILIC	+	Phenylalanine	165.0790	3.78	1	MID 28 [d]	ESI+ 166.0863, 149.060, 120.081, 103.0545, 79.055
HILIC	+	Leucine	131.0948	3.86	1	MID 24 [d]	ESI+ 132.1021, 118.086, 86.096, 69.07, 56.05, 44.049
HILIC	+	Isoleucine	131.0948	4.10	1	MID 23 [d]	ESI+ 132.1021, 118.086, 86.096, 56.05, 44.049
HILIC	+	Tryptophan	204.0900	3.91	1	MID 33 [d]	ESI+ 205.0973, 188.07, 146.059, 118.0652
HILIC	+	Valine	117.0792	4.71	1	MID 35 [d]	ESI+ 118.0865, 72.0820, 55.0559
HILIC	+	Tyrosine	181.0737	4.96	1	MID 34 [d]	ESI+ 182.091, 165.056, 147.046, 136.076, 123.045, 91.054
HILIC	+	Alanine	89.0481	5.51	1	MID 11 [d]	ESI+ 90.0557,44.0496
HILIC	+	Citrulline	175.0954	5.51	3	MID 16 [d]	ESI+ 176.1027, 159.0793, 134.076. 70.064
HILIC	-	88.0162@1.71	88.0162	1.71	4		No MSMS fragmentation
HILIC	-	198.0142@1.71	198.0142	1.71	4		ESI- 197.007, 162.838,107.327,86.491
HILIC	-	N-Acetylglycine	117.0424	2.59	2	MID 4230 [d]	ESI+ 118.0503, 76,0401, 72.0440; ESI- 116.0352, 74.0249
HILIC	-	2-Hydroxyethanesulfonate	125.9985	3.25	2		ESI- 124.9931,106.981,94.98, 79.9603
HILIC	-	Glutamate	147.0529	6.19	2	MID 19 [d]	ESI+148.0605,130.0503,84.0441;ESI- 146.0457,128.036,102.056
HILIC	-	491.1196@6.19	491.1196	6.19	4		No MSMS fragmentation

HILIC	-	Glutamate derivate316.0887@6.19	316.0887	6.19	3		No MSMS fragmentation
RP	+	Fatty acid 259.1608@7.63	259.1608	7.63	3		ESI+ 260.168, 218.936, 196.170, 181.157.
RP	+	Bile acid 386.2455 @8.21	386.2455	8.21	3		ESI+ 387.2582,369.237,351.223,261.185,105.072
RP	+	lysoPC(18:2)	519.3326	10.00	2	LMGP01050035[e]	ESI+ 520.3399, 184.072, 104.107; ESI- 564.3309,504.3116, 279.2330
RP	+	lysoPC(18:1)	521.3482	10.30	2	LMGP01050032 [e]	ESI+ 522.355, 184.072, 104.107; ESI- 566.346, 506.324. 281.2487
RP	+	lysoPC(p16:0)	479.3370	10.39	2	LMGP01070006 [e]	ESI+ 480.343, 184.073. 104.106; ESI- 524.336, 464.314, 375.230,168.042
RP	+	lysoPC(17:0)	509.3477	10.41	2	LMGP01050024 [e]	ESI+ 510.355, 184.073, 104.107, 88.1120; ESI- 554.346, 494.325, 269.248
RP	+	Fatty acid 364.333@10.46	364.333	10.46	3		ESI+ 365.3406,347.330
RP	+	lysoPC(19:1)	535.3647	10.46	2	MID 76572 [d]	ESI+ 536.376, 184.080,104.113; ESI- 580.363, 520.337, 295.263
RP	+	lysoPC(20:1)	549.3783	10.72	2	LMGP01050131 [e]	ESI+ 550.3855, 184.073, 104.106; ESI- 594.3820, 534.3553, 309.2795
RP	+	518.4333@12.07	518.4333	12.07	4		ESI+ 519.4406, 184.074,104.106,86.061
RP	+	PC(16:0/16:1)	731.5452	12.19	2	LMGP01011479 [e]	ESI+ 732.5525,184.072, 104.106, 86.0974 ; ESI- 776.5450,716.5227, 255.231, 253.2177
RP	+	DAG(16:1/16:1)	564.4744	12.72	2	LMGL02010011 [e]	ESI+ 582.5078, 547.472, 311.257
RP	+	DAG(14:0/16:0)	540.4754	13.12	1	LMGL02010378 [e]	ESI+ 558.5073, 523.471,313.273,285.242
RP	+	DAG 531.4484@13.29	531.4484	13.29	3		ESI + 549.4869, 60.081
RP	+	DAG 571.4437@13.30	571.4437	13.30	3		ESI + 589.4822, 74.097
RP	+	DAG(14:0/18:1)	566.4894	13.29	1	LMGL02010384 [e]	ESI+ 584.5235, 549.486, 339.288, 285.242
RP	+	590.4876@14.11	590.4876	14.11	4		No MSMS fragmentation
RP	+	DAG(16:0/18:1)	594.5202	14.32	2	LMGL02010307 [e]	ESI+ 612.5538, 577.5177, 339.288, 313.1123

RP	-	3-methyl-2-oxovaleric acid	130.0627	3.36	1	MID 122 [d]	ESI- 129.0559,83.0489, 57.3322, 41.0390
RP	-	428.2242@7.98	428.2242	7.98	4		ESI- 427.2167, 96.9591
RP	-	566.3105@8.28	566.3105	8.28	4		No MSMS fragmentation
RP	-	614.3679@8.60	614.3679	8.60	4		No MSMS fragmentation
RP	-	665.2645@9.87	665.2645	9.87	4		ESI- 664.2572, 604.234
RP	-	PC 583.3792@10.75	583.3792	10.75	3		No MSMS fragmentation
RP	-	597.4023@10.93	597.4023	10.93	4		ESI- 596.395, 536.372
RP	-	PC(16:1/14:0)	703.5004	11.76	2	LMGP01011475 [e]	ESI+ 704.5077, 184.072, 104.106; ESI- 748.5138, 688.494, 253.218, 227.202
RP	-	PC(15:1/18:2)	741.5433	12.61	2	LMGP01011444 [e]	ESI+ 742.555, 184.072, 104.106; ESI- 786.5672, 726.544, 279.2330,239.2015
RP	-	PC(17:0/18:2)	771.5533	12.70	2	LMGP01011505 [e]	ESI- 816.5723, 756.554, 279.233, 269.248

[a]RP: Reverse-phase chromatography; HILIC: hydrophilic interaction chromatography.

[b]'+' or ' –' denotes the electrospray ionization, positive or negative, respectively.

[c]Level of annotation confidence, according to the Metabolomics Standard Initiative reporting criteria.

[d]METLIN ID.

[e]Lipid Maps ID.

ESM Table 6. Odds ratio of type 2 diabetes according to metabolites in quartiles or per standard deviation (SD) among all cases and matched controls.

Metabolites	Model[a]	Q1(reference)[b]	Q2	Q3	Q4	OR per SD[c]	*p*[d]	*p*[e]
Lipids								
lysoPC(18:2)		5542145- 10402954	>10402954-12358218	>12358218-14829886	>14829886			
	Crude	1	0.51(0.34,0.76)	0.36(0.24,0.54)	0.15(0.1,0.23)	0.51(0.43,0.6)	<0.001	<0.001
	Model1	1	0.74(0.46,1.2)	0.58(0.36,0.95)	0.35(0.2,0.6)	0.72(0.6,0.87)	<0.0001	0.001
	Model2	1	0.78(0.47,1.31)	0.65(0.39,1.08)	0.33(0.18,0.58)	0.72(0.59,0.88)	<0.0001	0.002
	Model3	1	0.83(0.47,1.46)	0.56(0.32,1)	0.36(0.19,0.69)	0.75(0.6,0.94)	0.003	0.021
lysoPC(18:1)		8985288-15496102	>15496102-17503464	>17503464 - 19843689	>19843689			
	Crude	1	0.7(0.48,1.02)	0.46(0.32,0.67)	0.21(0.14,0.31)	0.56(0.48,0.65)	<0.001	<0.001
	Model1	1	0.85(0.54,1.34)	0.74(0.46,1.16)	0.45(0.27,0.75)	0.77(0.64,0.93)	0.002	0.007
	Model2	1	0.89(0.55,1.44)	0.76(0.47,1.24)	0.43(0.25,0.74)	0.77(0.63,0.93)	0.002	0.009
	Model3	1	0.88(0.51,1.51)	0.76(0.45,1.3)	0.36(0.2,0.66)	0.72(0.58,0.89)	0.003	0.008
lysoPC(p16:0)		269428.7 -732535.8	>732535.8 - 871491.2	>871491.2-1036619.6	>1036619.6			
	Crude	1	0.56(0.38,0.82)	0.4(0.27,0.58)	0.22(0.14,0.33)	0.55(0.47,0.64)	<0.001	<0.001
	Model1	1	0.62(0.39,0.99)	0.49(0.31,0.78)	0.39(0.24,0.63)	0.72(0.6,0.86)	<0.0001	0.001
	Model2	1	0.67(0.41,1.09)	0.54(0.33,0.87)	0.43(0.26,0.71)	0.74(0.61.0.89)	0.001	0.002
	Model3	1	0.75(0.44,1.27)	0.64(0.38,1.09)	0.5(0.29,0.86)	0.79(0.64.0.96)	0.023	0.034
lysoPC(17:0)		560744.4-1527794.0	>1527794.0-1866466.1	>1866466.1-2304555.8	>2304555.8			

	Crude	1	0.5(0.34,0.72)	0.41(0.28,0.6)	0.18(0.12,0.27)	0.52(0.44,0.61)	<0.001	<0.001
	Model1	1	0.64(0.41,1.01)	0.6(0.38.0.95)	0.37(0.22,0.61)	0.7(0.58,0.84)	<0.0001	<0.0001
	Model2	1	0.64(0.41,1.02)	0.62(0.38,1.01)	0.37(0.22,0.63)	0.7(0.57,0.85)	0.001	0.001
	Model3	1	0.74(0.43,1.26)	0.68(0.4,1.15)	0.43(0.24,0.75)	0.74(0.6,0.92)	0.009	0.013
lysoPC(19:1)		18585.57- 38158.04	>38158.04 - 49008.04	>49008.04- 59533.45	>59533.45-130090.87			
	Crude	1	0.52(0.35,0.78)	0.17(0.11,0.26)	0.15(0.1,0.24)	0.45(0.38,0.54)	<0.001	<0.001
	Model1	1	0.75(0.47,1.2)	0.26(0.16,0.44)	0.34(0.2,0.57)	0.63(0.51,0.77)	<0.0001	<0.0001
	Model2	1	0.79(0.48,1.3)	0.26(0.15,0.45)	0.34(0.2,0.59)	0.63(0.51,0.78)	<0.0001	<0.0001
	Model3	1	0.87(0.5,1.51)	0.26(0.15,0.48)	0.36(0.2,0.65)	0.64(0.51,0.81)	0.001	0.001
LysoPC(20:1)		44555.35- 153444.88	>153444.88- 193293.62	>193293.62- 241919.11	>241919.11			
	Crude	1	0.63(0.43,0.92)	0.46(0.31,0.67)	0.16(0.1,0.25)	0.49(0.41,0.58)	<0.001	<0.001
	Model1	1	0.88(0.56,1.39)	0.72(0.45,1.13)	0.38(0.23,0.63)	0.71(0.59,0.86)	<0.0001	0.001
	Model2	1	0.9(0.55,1.45)	0.73(0.45,1.19)	0.38(0.22,0.65)	0.72(0.6,0.88)	<0.0001	0.001
	Model3	1	0.91(0.54,1.53)	0.76(0.44,1.31)	0.44(0.24,0.8)	0.77(0.63,0.94)	0.011	0.021
PC(16:0/16:1)		311565.71 - 735329.92	> 735329.92 - 956905.9	>956905.91- 1228261.0	>1228261			
	Crude	1	1.74(1.2,2.53)	2.28(1.57,3.32)	3.57(2.39,5.34)	1.47(1.27,1.71)	<0.001	<0.001
	Model1	1	1.19(0.76,1.87)	1.55(0.98,2.46)	2.1(1.29,3.42)	1.24(1.05,1.47)	0.002	0.014
	Model2	1	1.29(0.81,2.06)	1.56(0.96,2.54)	2.13(1.28,3.54)	1.23(1.03,1.46)	0.004	0.027
	Model3	1	1.09(0.66,1.82)	1.38(0.8,2.36)	1.3(0.73,2.31)	1.02(0.84,1.24)	0.325	0.856
PC 583.3792@10.75		3822.86-14638.21	>14638.21- 18506.564	>18506.56- 23819.72	>23819.72			
	Crude	1	0.63(0.42,0.93)	0.27(0.18,0.42)	0.13(0.09,0.21)	0.41(0.34,0.5)	<0.001	<0.001
	Model1	1	0.69(0.44,1.1)	0.43(0.27,0.69)	0.28(0.16,0.47)	0.56(0.45,0.7)	<0.0001	<0.0001

	Model2	1	0.7(0.42,1.14)	0.41(0.25,0.67)	0.27(0.16,0.47)	0.55(0.44,0.7)	<0.0001	<0.0001
	Model3	1	0.71(0.41,1.21)	0.4(0.23,0.69)	0.29(0.16.0.53)	0.56(0.44,0.72)	<0.0001	<0.0001
PC(14:0/16:1)		1089.45 -10196.85	>10196.85-14258.06	14258.06-19964.92	>19964.89			
	Crude	1	1.49(1.04,2.14)	1.64(1.14,2.35)	1.79(1.25,2.58)	1.1(0.97,1.26)	<0.001	0.17
	Model1	1	1.36(0.87,2.13)	1.62(1.01,2.61)	1.53(0.97,2.43)	1.03(0.87,1.21)	0.084	0.740
	Model2	1	1.35(0.84,2.16)	1.54(0.94,2.53)	1.49(0.92,2.4)	1.02(0.85,1.21)	0.134	0.863
	Model3	1	1.16(0.69,1.97)	1.54(0.88,2.69)	0.97(0.56,1.68)	0.87(0.71,1.06)	0.888	0.219
PC(15:1/18:2)		8290.05-45566.01	>45566.01-55919.79	>55919.79-67668.31	>67668.31			
	Crude	1	0.54(0.37,0.81)	0.34(0.23,0.5)	0.18(0.12,0.27)	0.51(0.43,0.6)	<0.001	<0.001
	Model1	1	0.57(0.36,0.92)	0.44(0.27,0.72)	0.37(0.22,0.61)	0.7(0.58,0.85)	<0.0001	<0.0001
	Model2	1	0.56(0.34,0.92)	0.47(0.29,0.79)	0.4(0.23,0.68)	0.73(0.6,0.89)	0.001	0.002
	Model3	1	0.63(0.37,1.08)	0.57(0.33,1)	0.57(0.31,1.03)	0.84(0.68,1.04)	0.107	0.158
PC(17:0/18:2)		10047.53-34286.13	>34286.13-39831.56	>39831.56-46744.64	>46744.64			
	Crude	1	0.54(0.37,0.8)	0.31(0.2,0.46)	0.15(0.09,0.23)	0.48(0.41,0.57)	<0.001	<0.001
	Model1	1	0.79(0.5,1.25)	0.52(0.33,0.84)	0.34(0.2,0.6)	0.64(0.52,0.78)	<0.0001	<0.0001
	Model2	1	0.77(0.47,1.25)	0.5(0.3,0.83)	0.32(0.18,0.58)	0.63(0.51,0.77)	<0.0001	<0.0001
	Model3	1	0.86(0.51,1.45)	0.58(0.33,1)	0.42(0.22,0.79)	0.69(0.56,0.87)	0.007	0.007
DAG(16:1/16:1)		567.74-4887.39	>4887.39-6906.75	>6906.75-10192.85	>10192.85			
	Crude	1	2.02(1.37,2.99)	3.19(2.15,4.73)	5.25(3.45,7.98)	1.7(1.46,1.98)	<0.001	<0.001
	Model1	1	1.1(0.68,1.79)	1.69(1.06,2.69)	2.75(1.67,4.52)	1.37(1.15,1.63)	<0.0001	0.001
	Model2	1	1.04(0.63,1.71)	1.48(0.91,2.41)	2.54(1.52,4.26)	1.34(1.12,1.62)	<0.0001	0.002
	Model3	1	0.85(0.5,1.45)	1.03(0.59,1.79)	1.11(0.58,2.12)	0.88(0.69,1.13)	0.615	0.412

DAG(14:0/16:0)		429.36 -1562.302	>1562.30 - 2029.17	>2029.17 -2936.76	>2936.760			
	Crude	1	1.91(1.31,2.8)	3.21(2.18,4.72)	5.86(3.86,8.9)	1.9(1.57,2.29)	<0.001	<0.001
	Model1	1	1.38(0.88,2.15)	2.1(1.33,.331)	3.33(2.04,5.45)	1.5(1.23,1.81)	<0.0001	<0.0001
	Model2	1	1.27(0.79,2.05)	2.01(1.23,3.28)	3.18(1.89,5.34)	1.49(1.22,1.83)	<0.0001	<0.0001
	Model3	1	1.28(0.76,2.15)	1.63(0.94,2.81)	1.73(0.91,3.29)	1.11(0.88,1.39)	0.157	0.467
DAG 531.4484@13.29		1189.56-6421.47	>6421.48-8191.37	>8191.37-10963.03	>10963.03			
	Crude	1	2.54(1.67,3.86)	5.31(3.43,8.2)	7.75(4.92,12.2)	2.08(1.73,2.5)	<0.001	<0.001
	Model1	1	1.49(0.92,2.42)	2.66(1.6,4.44)	3.72(2.2,6.27)	1.58(1.3,1.92)	<0.0001	<0.0001
	Model2	1	1.6(0.96,2.66)	2.56(1.47,4.46)	3.77(2.19,6.49)	1.57(1.28,1.92)	<0.0001	<0.0001
	Model3	1	1.42(0.82,2.47)	2.18(1.17,4.06)	2.05(1.02,4.11)	1.12(0.86,1.47)	0.108	0.468
DAG 571.4437@13.30		6679.13-12083.56	>12083.56-15865.96	>15865.96-24172.84	>24172.83-116762.75			
	Crude	1	1.92(1.3,2.85)	3.58(2.35,5.44)	7.91(4.92,12.72)	2.05(1.69,2.49)	<0.001	<0.001
	Model1	1	1.71(1.06,2.76)	2.28(1.35,3.85)	4.84(2.72,8.59)	1.61(1.32,1.96)	<0.0001	<0.0001
	Model2	1	1.64(0.99,2.73)	2.11(1.21,3.65)	4.79(2.64,8.69)	1.61(1.3,1.98)	<0.0001	<0.0001
	Model3	1	1.49(0.86,2.56)	1.79(0.97,3.3)	2.52(1.22,5.22)	1.1(0.83,1.45)	0.039	0.559
DAG(14:0/18:1)		8938.23- 29784.781	>29784.78-43950.68	>43950.68 - 70629.29	>70629.29			
	Crude	1	2.3(1.53,3.47)	4.67(3.05,7.13)	7.06(4.51,11.07)	2.06(1.71,2.47)	<0.001	<0.001
	Model1	1	1.42(0.88,2.29)	2.38(1.44,3.92)	3.31(1.97,5.58)	1.59(1.31,1.92)	<0.0001	<0.0001
	Model2	1	1.41(0.86,2.31)	2.16(1.27,3.66)	3.23(1.89,5.53)	1.58(1.29,1.93)	<0.0001	<0.0001
	Model3	1	1.24(0.72,2.13)	1.74(0.96,3.16)	1.68(0.84,3.36)	1.13(0.86,1.48)	0.207	0.451
DAG(16:0/18:1)		53225.58 - 237378.31	>237378.31-361759.53	>361759.53 - 544545.56	>544545.56			

	Crude	1	2.41(1.59,3.64)	4.64(2.93,7.35)	9.44(5.79,15.39)	2.42(1.94,3.02)	<0.001	<0.001
	Model1	1	1.73(1.07,2.78)	2.61(1.52,4.49)	4.39(2.5,7.72)	1.73(1.38,2.17)	<0.0001	<0.0001
	Model2	1	1.69(1.03,2.77)	2.46(1.39,4.37)	4.4(2.43,7.97)	1.73(1.36,2.19)	<0.0001	<0.0001
	Model3	1	1.66(0.98,2.82)	2.36(1.24,4.5)	2.44(1.16,5.11)	1.2(0.88,1.64)	0.084	0.330
Fatty acid 364.333@10.46		4674.69-10592.64	>10592.65-12296.52	> 12296.52-13963.6	>13963.60			
	Crude	1	1.99(1.35,2.93)	3.16(2.12,4.71)	4.71(3.09,7.2)	2.01(1.7,2.38)	<0.001	<0.001
	Model1	1	1.28(0.81,2.02)	1.59(0.98,2.57)	2.4(1.45,3.97)	1.52(1.25,1.84)	0.001	<0.0001
	Model2	1	1.23(0.76,2.01)	1.53(0.92,2.54)	2.28(1.34,3.87)	1.52(1.24,1.87)	0.002	<0.0001
	Model3	1	1.18(0.7,1.98)	1.38(0.8,2.39)	1.99(1.12,3.55)	1.43(1.14,1.79)	0.027	0.007
Fatty acid 259.1608@7.63		111.55 -1227.24	>1227.24 - 1650.71	> 1650.71 - 2252.28	>2252.28			
	Crude	1	1.91(1.21,3.01)	3.57(2.23,5.72)	10.99(6.71,18.01)	3.83(2.8,5.24)	<0.001	<0.001
	Model1	1	1.52(0.9,2.58)	2.49(1.45,4.26)	4.55(2.57,8.06)	2.07(1.52,2.82)	<0.0001	<0.0001
	Model2	1	1.49(0.85,2.62)	2.71(1.52,4.83)	4.74(2.56,8.79)	2.07(1.5,2.86)	<0.0001	<0.0001
	Model3	1	1.41(0.78,2.55)	2.29(1.24,4.23)	3.15(1.63,6.09)	1.82(1.3,2.53)	0.002	0.003
Non-lipids								
2-Methylbutyroylcarnitine		44899.72 - 124213.18	>124213.18 - 153260.41	>153260.41 - 189182.32	>189182.32			
	Crude	1	1.74(1.18,2.57)	2.81(1.86,4.26)	5.53(3.57,8.57)	1.03(0.9,1.18)	<0.001	0.65
	Model1	1	1.24(0.78,1.99)	1.39(0.84,2.29)	2.65(1.58,4.44)	0.97(0.84,1.11)	<0.0001	0.637
	Model2	1	1.25(0.76,2.04)	1.37(0.8,2.36)	2.88(1.66,4.99)	0.98(0.85,1.13)	<0.0001	0.781
	Model3	1	1.1(0.65,1.88)	1.43(0.79,2.59)	3.15(1.71,5.79)	1.01(0.87,1.17)	0.001	0.930
3-Hydroxyisovalerylcarnitine		959.87 -3358.91	>3358.91-4085.34	>4085.34 -5066.69	>5066.69			
	Crude	1	1.42(0.98,2.05)	2.17(1.48,3.18)	3.41(2.28,5.1)	1.78(1.52,2.1)	<0.001	<0.001

	Model1	1	0.98(0.62,1.55)	1.56(0.98,2.5)	2.11(1.3,3.41)	1.46(1.21,1.77)	0.001	<0.0001
	Model2	1	0.83(0.51,1.35)	1.5(0.92,2.47)	2.09(1.25,3.48)	1.46(1.19,1.78)	0.001	<0.0001
	Model3	1	0.95(0.56,1.61)	1.79(1.04,3.1)	2.31(1.29,4.13)	1.43(1.15,1.79)	0.004	0.007
Phenylalanine		930182.1-1465793.9	>1465793.9-1628410.2	>1628410.2-1810575.3	>1810575.3			
	Crude	1	2.02(1.37,2.98)	3.29(2.18,4.97)	4.37(2.86,6.69)	1.71(1.45,2)	<0.001	<0.001
	Model1	1	1.32(0.83,2.1)	1.65(1.01,2.69)	1.89(1.15,3.11)	1.24(1.03.1.48)	0.011	0.022
	Model2	1	1.31(0.81,2.13)	1.68(0.99,2.87)	1.79(1.05,3.05)	1.2(0.99.1.46)	0.031	0.068
	Model3	1	1.28(0.75,2.18)	1.59(0.9,2.79)	1.53(0.85,2.76)	1.18(0.96,1.45)	0.164	0.172
Leucine		1755094-3290315	>3290315-3928437	>3928437-4585153	>4585153			
	Crude	1	3.17(2.1,4.79)	4.83(3.12,7.46)	7.64(4.73,12.33)	2.28(1.9,2.74)	<0.001	<0.001
	Model1	1	2.16(1.32,3.53)	2.35(1.41.3.93)	3.5(1.98.6.17)	1.66(1.34.2.05)	<0.0001	<0.0001
	Model2	1	2.17(1.31,3.62)	2.25(1.31.3.88)	3.46(1.88.6.36)	1.64(1.3.2.06)	<0.0001	<0.0001
	Model3	1	2.78(1.59,4.84)	2.31(1.25.4.27)	3.63(1.83.7.19)	1.63(1.26.2.11)	0.006	0.001
Isoleucine		1755094-3290315	548262.8-1055445.6	>1055445.6-1293007.4	>1293007.4-1553874.5	>1553874.5		
	Crude	1	3.66(2.43,5.52)	6.15(3.9,9.69)	10.62(6.45,17.51)	2.47(2.04,2.99)	<0.001	<0.001
	Model1	1	2.62(1.61,4.26)	3.23(1.93,5.42)	4.74(2.68,8.36)	1.79(1.44,2.21)	<0.0001	<0.0001
	Model2	1	2.78(1.67,4.62)	3.27(1.88,5.7)	4.83(2.61,8.92)	1.78(1.41,2.24)	<0.0001	<0.0001
	Model3	1	3.05(1.74,5.35)	3.36(1.8,6.26)	4.43(2.2,8.91)	1.7(1.31,2.21)	0.001	0.001
Valine		1015669-1525380	>1525380-1692882	>1692882-1892817	>1892817			
	Crude	1	2.46(1.7,3.55)	3.79(2.55,5.63)	4.54(3.02,6.84)	1.61(1.37,1.89)	<0.001	<0.001
	Model1	1	1.86(1.19,2.9)	2.01(1.24,3.24)	2.31(1.4,3.81)	1.26(1.05,1.51)	0.003	0.013
	Model2	1	2.01(1.24,3.24)	2.05(1.23,3.41)	2.23(1.3,3.84)	1.22(1,1.47)	0.014	0.054

	Model3	1	2.14(1.26,3.63)	2.27(1.29,3.98)	2.59(1.4,4.77)	1.31(1.05,1.62)	0.016	0.026
Tryptophan		889336.5-1676979.4	>1676979.4-1902695.8	>1902695.8-2126727.0	>2126727.0			
	Crude	1	2.34(1.58,3.47)	2.81(1.86,4.22)	4.41(2.87,6.8)	1.66(1.42,1.95)	<0.001	<0.001
	Model1	1	1.9(1.16,3.11)	2.72(1.62,4.56)	3.63(2.12,6.22)	1.53(1.26,1.85)	<0.0001	<0.0001
	Model2	1	2.07(1.22,3.51)	3.06(1.77,5.29)	3.81(2.15,6.78)	1.52(1.24,1.86)	<0.0001	<0.0001
	Model3	1	2.22(1.25,3.96)	3.5(1.92,6.39)	3.87(2.03,7.4)	1.55(1.24,1.95)	0.001	0.001
L-Tyrosine		114519.6 -204586.5	>204586.5-240327.9	>240327.9-288241.1	> 288241.1			
	Crude	1	3.22(2.08,5)	5.82(3.66,9.24)	9.7(5.93,15.86)	2.33(1.95,2.79)	<0.001	<0.001
	Model1	1	2.39(1.45,3.93)	3.17(1.87,5.36)	3.5(1.97,6.2)	1.56(1.26,1.92)	<0.0001	<0.0001
	Model2	1	2.46(1.44,4.21)	3.23(1.83,5.71)	3.28(1.79,6.01)	1.51(1.21,1.88)	0.001	<0.0001
	Model3	1	2.16(1.22,3.82)	2.82(1.54,5.16)	2.47(1.28,4.77)	1.44(1.13,1.84)	0.025	0.010
Alanine		190586.9 -368299.1	>368299.1 - 452178.3	>452178.3 - 547506.1	>547506.1			
	Crude	1	1.74(1.2,2.53)	2.8(1.93,4.06)	3.77(2.54,5.6)	1.71(1.48,1.98)	<0.001	<0.001
	Model1	1	1.23(0.78,1.94)	1.79(1.15,2.81)	2.5(1.55,4.05)	1.48(1.24,1.75)	<0.0001	<0.0001
	Model2	1	1.13(0.7,1.81)	1.68(1.04,2.72)	2.4(1.45,3.99)	1.46(1.22,1.74)	<0.0001	<0.0001
	Model3	1	1.01(0.61,1.69)	1.36(0.81,2.3)	1.8(1.01,3.19)	1.3(1.06,1.58)	0.030	0.021
Citrulline		1140.15-4471.79	>4471.79-5742.02	>5742.02-7524.77	>7524.77			
	Crude	1	1.45(0.99,2.14)	1.7(1.16,2.49)	3.58(2.37,5.39)	1.68(1.42,1.98)	<0.001	<0.001
	Model1	1	1.69(1.04,2.76)	1.69(1.04,2.74)	2.54(1.53,4.22)	1.46(1.21,1.76)	0.001	<0.0001
	Model2	1	1.72(1.03,2.86)	1.6(0.97,2.63)	2.53(1.47,4.34)	1.45(1.19,1.76)	0.003	<0.0001
	Model3	1	1.39(0.81,2.38)	1.53(0.9,2.63)	2.21(1.24,3.93)	1.4(1.13,1.73)	0.016	0.007
N-Acetylglycine		12934.19 -24194.09	>24194.09 - 30057.79	>30057.79 - 40192.87	>40192.87			

	Crude	1	0.83(0.56,1.23)	0.42(0.28,0.61)	0.24(0.16,0.35)	0.52(0.44,0.63)	<0.001	<0.001
	Model1	1	1.02(0.64,1.64)	0.58(0.36,0.94)	0.4(0.25,0.65)	0.68(0.57,0.83)	<0.0001	<0.0001
	Model2	1	0.91(0.55,1.49)	0.54(0.33,0.9)	0.37(0.23,0.62)	0.69(0.57,0.84)	<0.0001	<0.0001
	Model3	1	0.88(0.51,1.52)	0.54(0.31,0.94)	0.52(0.29,0.91)	0.81(0.66,0.99)	0.03	0.055
2-Hydroxyethanesulfonate		48375.95-159379.99	> 159379.99-185079.76	>185079.76-215290.61	>215290.61			
	Crude	1	0.53(0.37,0.76)	0.41(0.28,0.59)	0.3(0.2,0.44)	0.62(0.53,0.71)	<0.001	<0.001
	Model1	1	0.75(0.49,1.17)	0.66(0.42,1.03)	0.45(0.28,0.73)	0.73(0.61,0.87)	0.001	0.001
	Model2	1	0.77(0.48,1.22)	0.7(0.43,1.12)	0.42(0.25,0.71)	0.71(0.59,0.86)	0.001	0.001
	Model3	1	0.78(0.47,1.29)	0.73(0.43,1.23)	0.5(0.28,0.89)	0.76(0.62,0.94)	0.031	0.021
Glutamate		14385.209 - 22435.910	>22435.91 - 32759.97	> 32759.97-49842.53	>49842.53			
	Crude	1	1.59(1.08,2.33)	2.21(1.52,3.22)	3.62(2.43,5.4)	1.56(1.34,1.81)	<0.001	<0.001
	Model1	1	1.22(0.76,1.97)	1.37(0.86,2.19)	1.66(1.2,75)	1.13(0.95,1.35)	0.055	0.170
	Model2	1	1.16(0.71,1.9)	1.24(0.76,2.02)	1.48(0.87,2.51)	1.1(0.91,1.33)	0.157	0.340
	Model3	1	1.21(0.7,2.09)	1.04(0.61,1.77)	1.2(0.66,2.16)	0.99(0.8,1.23)	0.712	0.930
Glutamate derivate 316.0887@6.19		807.4464-4650.7936	>4650.79 - 7310.00	>7310.00-12023.42	>12023.42			
	Crude	1	2(1.35,2.95)	2.95(1.98,4.39)	4.57(3.01,6.92)	1.71(1.43,2.03)	<0.001	<0.001
	Model1	1	1.49(0.93,2.39)	1.62(1,2.61)	2.17(1.3,3.63)	1.22(1.01,1.47)	0.008	0.045
	Model2	1	1.53(0.93,2.52)	1.51(0.91,2.52)	2.15(1.25,3.71)	1.2(0.98,1.46)	0.015	0.078
	Model3	1	1.45(0.84,2.5)	1.24(0.71,2.16)	1.55(0.86,2.82)	1.08(0.86,1.35)	0.292	0.559
Bile acid386.2455 @8.21		5682-15332	>15332.67-20755.82	>20755.82 - 29443.22	>29443.22			
	Crude	1	2.36(1.57,3.56)	3.08(2.06,4.61)	9.37(5.83,15.07)	2.62(2.1,3.27)	<0.001	<0.001
	Model1	1	1.66(1.04,2.65)	1.59(1,2.55)	3.66(2.13,6.27)	1.71(1.36,2.16)	<0.0001	<0.0001

	Model2	1	1.75(1.07,2.85)	1.58(0.96,2.6)	3.46(1.97,6.07)	1.68(1.32,2.14)	<0.0001	<0.0001
	Model3	1	1.63(0.95,2.8)	1.59(0.92,2.73)	2.97(1.59,5.55)	1.53(1.17,2.01)	0.004	0.007
3-methyl-2-oxovaleric acid		647131-1141859	>1141859.1-1334329.1	> 1334329.1-1580743.4	>1580743.4			
	Crude	1	2.3(1.55,3.41)	3.62(2.35,5.58)	7.43(4.57,12.09)	2.18(1.8,2.64)	<0.001	<0.001
	Model1	1	1.92(1.18,3.13)	3.36(1.96,5.77)	4.79(2.66,8.62)	1.8(1.43,2.27)	<0.0001	<0.0001
	Model2	1	2.18(1.29,3.69)	3.71(2.1,6.55)	6.05(3.17,11.53)	1.94(1.51,2.49)	<0.0001	<0.0001
	Model3	1	2.63(1.47,4.71)	4.76(2.49,9.09)	6.41(3.08,13.34)	1.9(1.44.2.51)	<0.0001	<0.0001
Unknown compounds								
161.0062@2.7		1050.38- 2883.62	>2883.62-3977.08	> 3977.08 - 5553.36	>5553.36			
	Crude	1	0.6(0.41,0.89)	0.37(0.25,0.54)	0.23(0.15,0.34)	0.52(0.43,0.62)	<0.001	<0.001
	Model1	1	0.71(0.44,1.14)	0.48(0.3,0.76)	0.39(0.24,0.62)	0.67(0.55,0.81)	<0.0001	<0.0001
	Model2	1	0.67(0.41,1.11)	0.48(0.3,0.79)	0.38(0.23,0.63)	0.67(0.55,0.82)	<0.0001	<0.0001
	Model3	1	0.69(0.41,1.18)	0.5(0.29,0.85)	0.55(0.32,0.96)	0.78(0.64,0.96)	0.061	0.034
88.0162@1.71		75088-178342	>178342.79-220623.96	>220623.96-272121.32	>272121.32			
	Crude	1	1.07(0.74,1.57)	2(1.4,2.86)	4.11(2.76,6.12)	1.86(1.59,2.17)	<0.001	<0.001
	Model1	1	0.85(0.54,1.35)	1.22(0.78,1.9)	2.47(1.52,4)	1.51(1.25,1.81)	<0.0001	<0.0001
	Model2	1	0.79(0.49,1.28)	1.09(0.68,1.75)	2.65(1.58,4.44)	1.53(1.26,1.87)	<0.0001	<0.0001
	Model3	1	0.71(0.42,1.2)	0.97(0.57,1.64)	2.13(1.2,3.78)	1.41(1.13,1.76)	0.007	0.007
198.0142@1.71		9455.46-25208.93	>25208.93-32759.25	>32759.25-40880.590	>40880.59			
	Crude	1	1.19(0.83,1.72)	1.68(1.17,2.41)	4.01(2.71,5.94)	1.78(1.53,2.07)	<0.001	<0.001
	Model1	1	0.84(0.53,1.33)	0.89(0.57,1.39)	2.25(1.4,3.61)	1.43(1.19,1.71)	0.001	<0.0001
	Model2	1	0.8(0.49,1.3)	0.86(0.54,1.38)	2.35(1.41,3.91)	1.46(1.2,1.77)	0.001	<0.0001

	Model3	1	0.73(0.43,1.24)	0.68(0.39,1.16)	2.04(1.16,3.57)	1.36(1.1,1.67)	0.020	0.011
491.1196@6.19		1046.11 -3371.72	>3371.72 - 4472.63	>4472.63 -5759.09	>5759.09			
	Crude	1	0.63(0.43,0.92)	0.51(0.36,0.75)	0.26(0.18,0.39)	0.57(0.49,0.66)	<0.001	<0.001
	Model1	1	0.99(0.62,1.58)	0.91(0.58,1.42)	0.6(0.37,0.97)	0.77(0.65,0.92)	0.026	0.005
	Model2	1	1.11(0.68,1.81)	1(0.62,1.61)	0.58(0.35,0.94)	0.75(0.62,0.9)	0.018	0.002
	Model3	1	1.21(0.71,2.04)	1.11(0.66,1.88)	0.73(0.42,1.26)	0.81(0.66,1)	0.212	0.074
518.4333@12.07		101.99-787.14	>787.14-1464.06	>1464.06-2549.52	>2549.52			
	Crude	1	1.61(1.05,2.47)	3.05(1.91,4.88)	7.75(4.53,13.28)	1.86(1.51,2.28)	<0.001	<0.001
	Model1	1	1.74(1.03,2.94)	2.4(1.35,4.27)	5.58(2.9,10.74)	1.54(1.24,1.91)	<0.0001	<0.0001
	Model2	1	1.72(1,2.95)	2.5(1.37,4.58)	5.3(2.66,10.55)	1.54(1.22,1.93)	<0.0001	<0.0001
	Model3	1	1.77(0.98,3.21)	1.99(1.02,3.89)	2.76(1.23,6.18)	1.1(0.84,1.45)	0.046	0.552
590.4876@14.11		4231.08- 8756.28	>8756.28-10451.07	>10451.07-13989.35	>13989.35			
	Crude	1	1.94(1.28,2.92)	3.48(2.23,5.41)	6.7(4.18,10.74)	1.97(1.6,2.44)	<0.001	<0.001
	Model1	1	1.45(0.89,2.36)	2.26(1.33,3.87)	3.97(2.26,6.98)	1.5(1.22,1.85)	<0.0001	<0.0001
	Model2	1	1.52(0.9,2.56)	2.3(1.3,4.08)	4.08(2.27,7.35)	1.5(1.21,1.84)	<0.0001	<0.0001
	Model3	1	1.43(0.81,2.51)	1.73(0.93,3.21)	2.28(1.15,4.5)	1.14(0.9,1.46)	0.046	0.355
428.2242@7.98		1001.47 -10422.77	>10422.77 - 18026.560	>18026.56-30006.01	>30006.01			
	Crude	1	1.61(1.09,2.37)	3.58(2.37,5.42)	8.25(5.27,12.92)	2.49(2.04,3.03)	<0.001	<0.001
	Model1	1	1.07(0.68,1.68)	1.83(1.13,2.96)	2.9(1.72,4.88)	1.68(1.35,2.09)	<0.0001	<0.0001
	Model2	1	1.03(0.64,1.67)	1.91(1.15,3.17)	3.37(1.92,5.91)	1.76(1.39,2.21)	<0.0001	<0.0001
	Model3	1	0.88(0.52,1.49)	1.69(0.96,2.96)	2.6(1.4,4.8)	1.64(1.28,2.1)	0.001	0.001
566.3105@8.28		156.42 -1259.79	>1259.79 - 1867.03	>1867.03 -2603.58	>2603.58			

	Crude	1	1.73(1.17,2.54)	2.95(2,4.34)	5.89(3.9,8.9)	2.22(1.84,2.68)	<0.001	<0.001
	Model1	1	1.58(0.99,2.52)	1.69(1.06,2.7)	3.3(1.98,5.51)	1.67(1.35,2.07)	<0.0001	<0.0001
	Model2	1	1.53(0.93,2.5)	1.72(1.05,2.83)	3.43(1.99,5.94)	1.69(1.34,2.13)	<0.0001	<0.0001
	Model3	1	1.19(0.7,2.04)	1.42(0.84,2.42)	2.48(1.35,4.56)	1.45(1.13,1.87)	0.006	0.010
614.3679@8.60		2400.232 -7632.345	>7632.34-10044.63	>10044.63-13350.02	>13350.02-50832.42			
	Crude	1	1.9(1.29,2.79)	3.48(2.35,5.14)	4.81(3.22,7.2)	1.98(1.67,2.36)	<0.001	<0.001
	Model1	1	1.48(0.93,2.35)	2.46(1.55,3.93)	2.52(1.54,4.14)	1.55(1.27,1.89)	<0.0001	<0.0001
	Model2	1	1.38(0.85,2.24)	2.36(1.46,3.81)	2.19(1.29,3.71)	1.48(1.2,1.82)	0.002	<0.0001
	Model3	1	1.46(0.86,2.47)	2.09(1.24,3.53)	1.88(1.03,3.45)	1.35(1.07,1.71)	0.041	0.021
665.2645@9.87		13032.68-21474.87	>21474.87-25216.80	>25216.80-28567.48	>28567.48			
	Crude	1	0.79(0.53,1.18)	0.35(0.23,0.53)	0.22(0.15,0.34)	0.52(0.45,0.61)	<0.001	<0.001
	Model1	1	1.09(0.68,1.77)	0.56(0.34.0.91)	0.51(0.31.0.85)	0.72(0.59.0.86)	0.001	0.001
	Model2	1	1.14(0.69,1.9)	0.55(0.32.0.92)	0.51(0.29.0.87)	0.7(0.57.0.85)	0.001	0.001
	Model3	1	1.19(0.68,2.07)	0.5(0.28.0.89)	0.59(0.33.1.06)	0.75(0.61.0.93)	0.02	0.02
597.4023@10.93		3056.48 -9224.37	>9224.36-11668.18	>11668.18-14492.96	>14492.96-58146.11			
	Crude	1	0.58(0.39,0.85)	0.27(0.18,0.4)	0.15(0.1,0.23)	0.47(0.39,0.57)	<0.001	<0.001
	Model1	1	0.71(0.45,1.11)	0.43(0.27,0.7)	0.34(0.2,0.58)	0.71(0.57,0.88)	<0.0001	0.002
	Model2	1	0.76(0.47,1.22)	0.44(0.26,0.74)	0.34(0.19,0.59)	0.7(0.56,0.88)	<0.0001	0.003
	Model3	1	0.76(0.45,1.29)	0.44(0.25,0.78)	0.38(0.2,0.7)	0.72(0.57,0.92)	0.004	0.019

[a]Crude model, no adjustments; Model 1, adjusted for fasting glucose and BMI ; Model 2, further adjusted for physical activity, education, smoking, consumption of alcohol, dietary fibre, red and processed meat, and coffee; Model 3, further adjusted for plasma total cholesterol, triacylglycerols and systolic and diastolic BP.

[b]Q1-Q4 represent the quartiles for each of metabolites.

[c]For each metabolite. ORs were calculated for quartiles or per SD increment (mean=0, SD=1).

[d]False discovery rate adjusted *p* values for trend across quartiles are presented.

[e]False discovery rate adjusted *p* values for OR per SD are presented.

ESM Table 7. Effect of medication on changes in metabolites levels between baseline and 10 year follow up among cases (n=187).

	Baseline vs Follow up			
Metabolites	No medication	Antidiabetic only	Others	Has medication
DAG(14:0/16:0)	Higher			
DAG(14:0/18:1)	Higher			
DAG(16:0/18:1)	Higher			
PC(16:0/16:1)	Higher			
Phenylalanine	Higher			
Tryptophan				Lower
161.0062@2.7	Lower			
N-Acetylglycine	Lower		Higher	
2-Hydroxyethanesulfonate			Higher	
428.2242@7.98	Higher			
597.4023@10.93		Higher		
HOMA-B%		Lower		Lower
HOMA-IR		Higher	Higher	Higher

The difference between baseline and after 10 years follow up among type 2 diabetes cases, assessed by paired t test (p<0.05).'High' means metabolites level is higher at follow up than at baseline, and vice versa.

ESM Figures

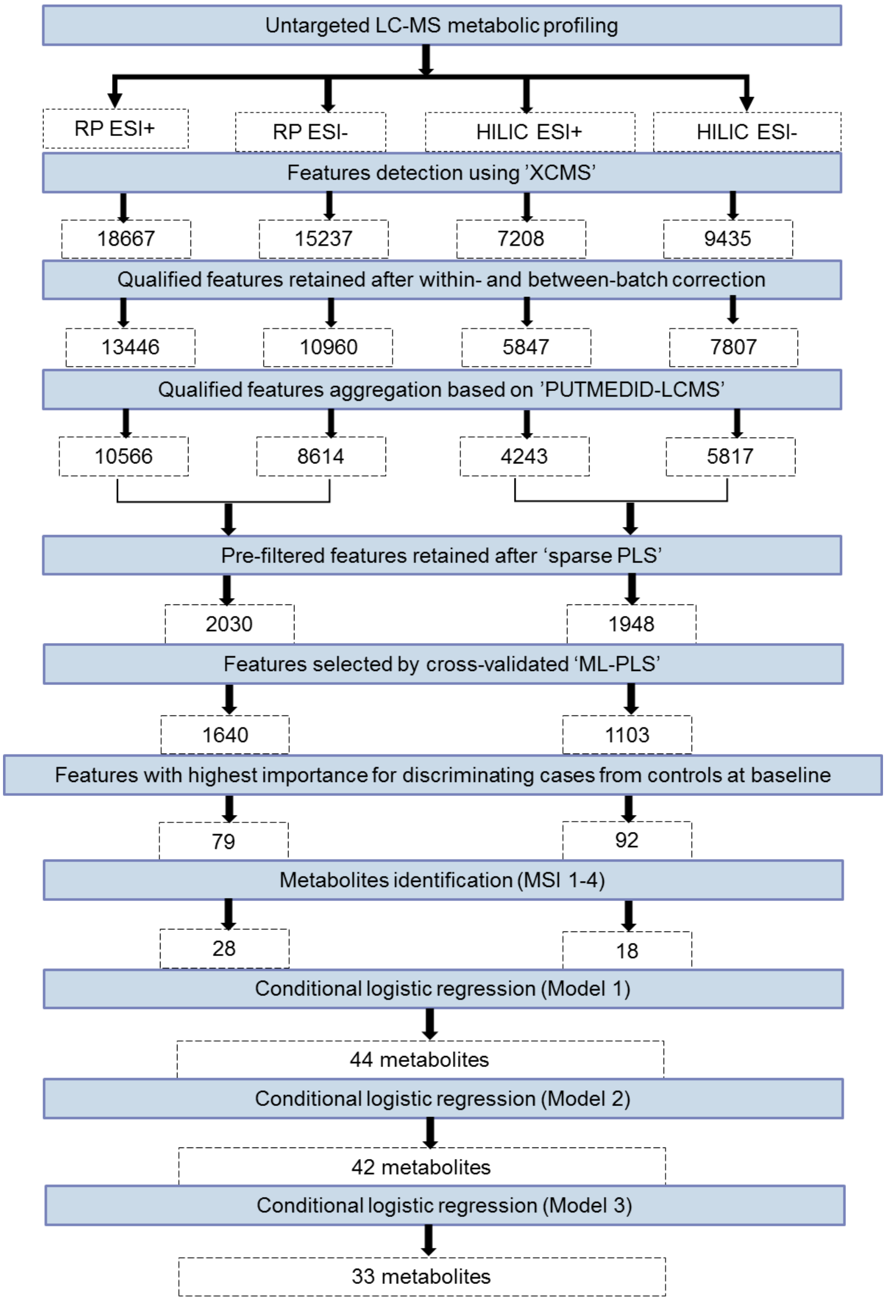

ESM Fig. 1. Overall workflow for selection of metabolites associated with odds of developing type 2 diabetes. Number of features or metabolites at each step is presented. Model 1: adjustment for fasting plasma glucose and BMI; Model 2: further adjustment for physical activity, education, smoking, and consumption of alcohol, dietary fibre, coffee and red and processed meat; Model 3: additionally adjustment for plasma total cholesterol, triacyglycerol, and systolic and diastolic BP.

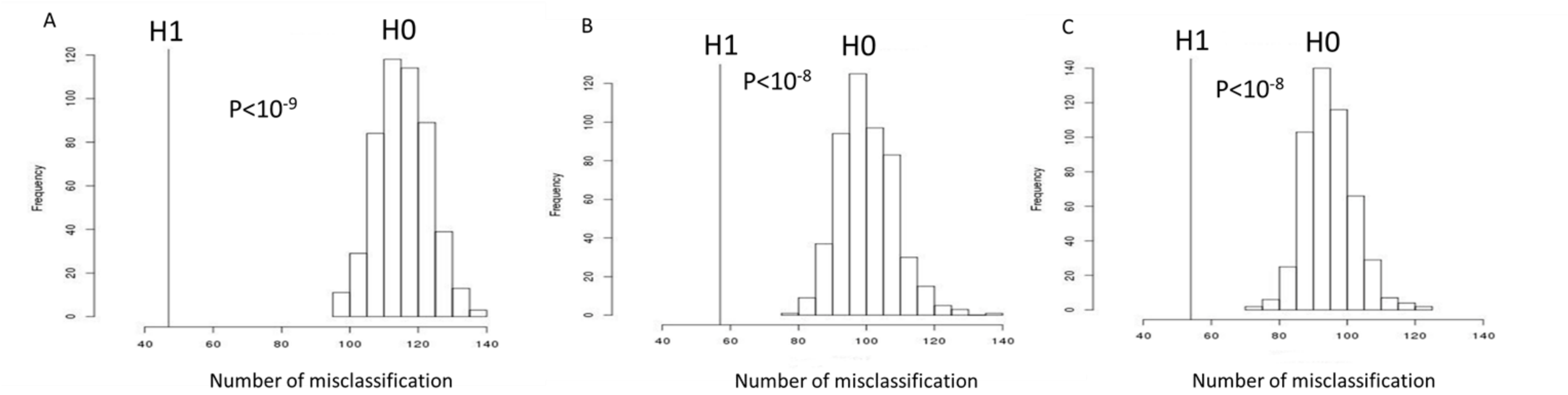

ESM Fig. 2 Test for performance of multilevel partial least squares classification on metabolomics data obtained from: A, hydrophilic interaction chromatography data including hexose signals; B, HILIC data excluding hexose signals; C, Reverse-phase chromatography data, versus random permutations. H1 represents the misclassification for actual model and H0 is the distribution of random permutation. All actual models outperformed random permutations, as assessed by one tailed student’s t test.

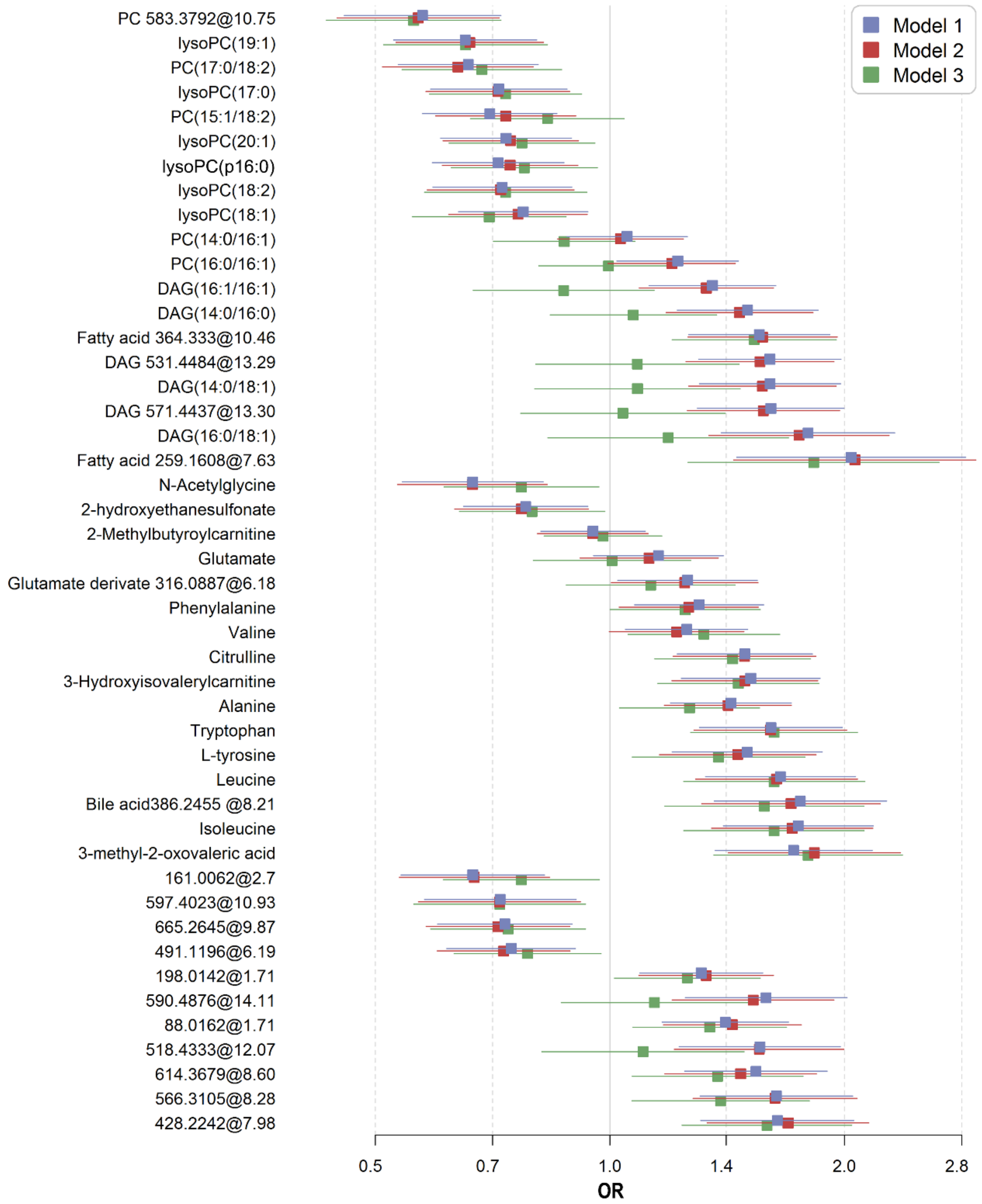

ESM Fig. 3 OR per SD increment (95% CI) of metabolites in the sensitivity analysis after excluding 95 pairs of participants where cases had abnormal fasting glucose (≥5.9 mmol/l) and 2h-plasma glucose(>11.1 mmol/l) at baseline or developed diabetes during the first two years after baseline sampling. Model 1(blue): adjusting for fasting glucose, and BMI; model 2 (red): further adjusting for physical activity, education, smoking, and consumption of alcohol, dietary fibre, coffee and red and processed meat; model 3 (green): additionally adjusting for plasma total cholesterol, triacylglycerol, systolic and diastolic BP.

Lipids

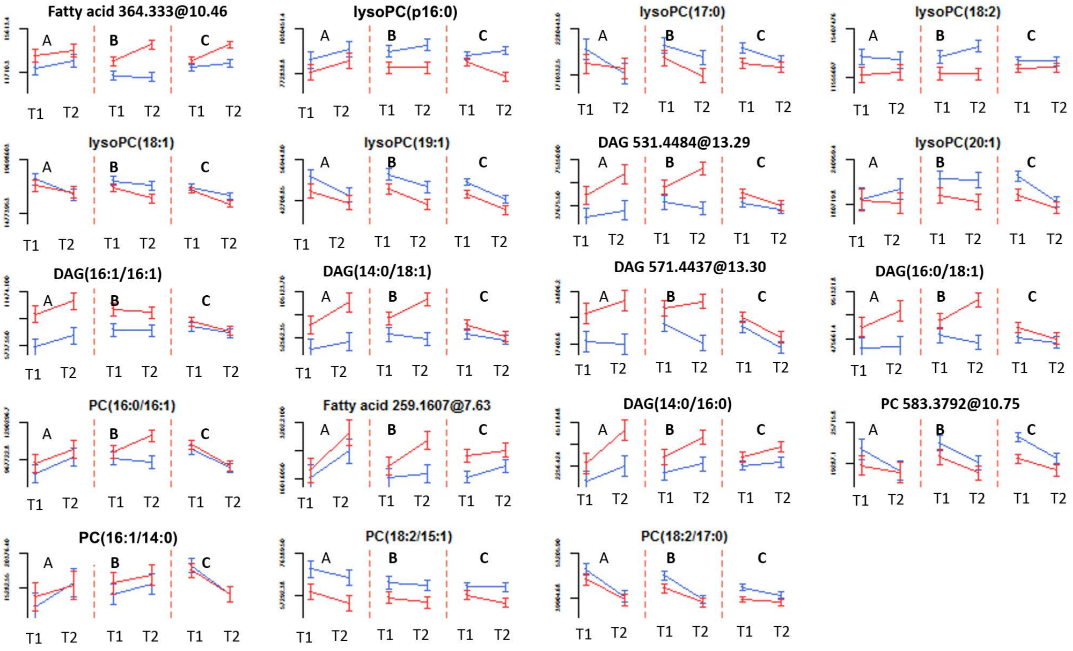

Non- lipids

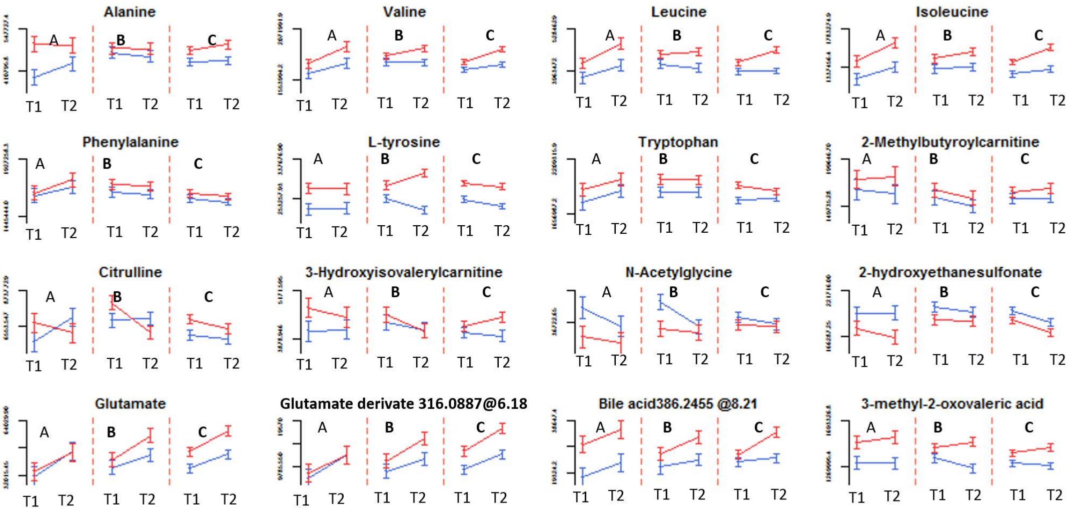

Unknown metabolites

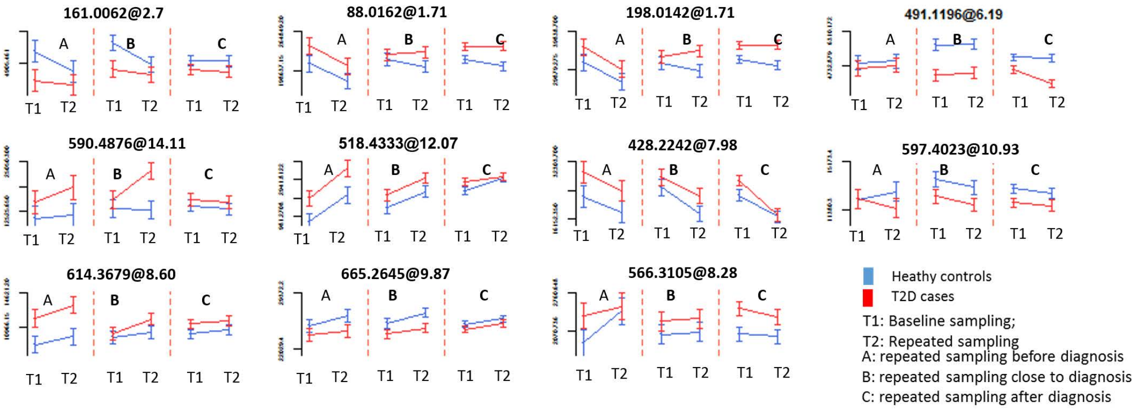

ESM Fig. 4 Changes in metabolites over time among individuals who later developed type 2 diabetes (n=187). Values are presented as least square means ± standard error obtained from mixed models. Group A: time of second sampling occurred before time of type 2 diabetes diagnosis (n=26 pairs); group B: time of second sampling occurred within six months from time of type 2 diabetes diagnosis (n=52 pairs); group C: time of type 2 diabetes diagnosis occurred after second sampling (n=109 pairs). T1 denotes sampling at baseline and T2 denotes second sampling after 10 years follow up.

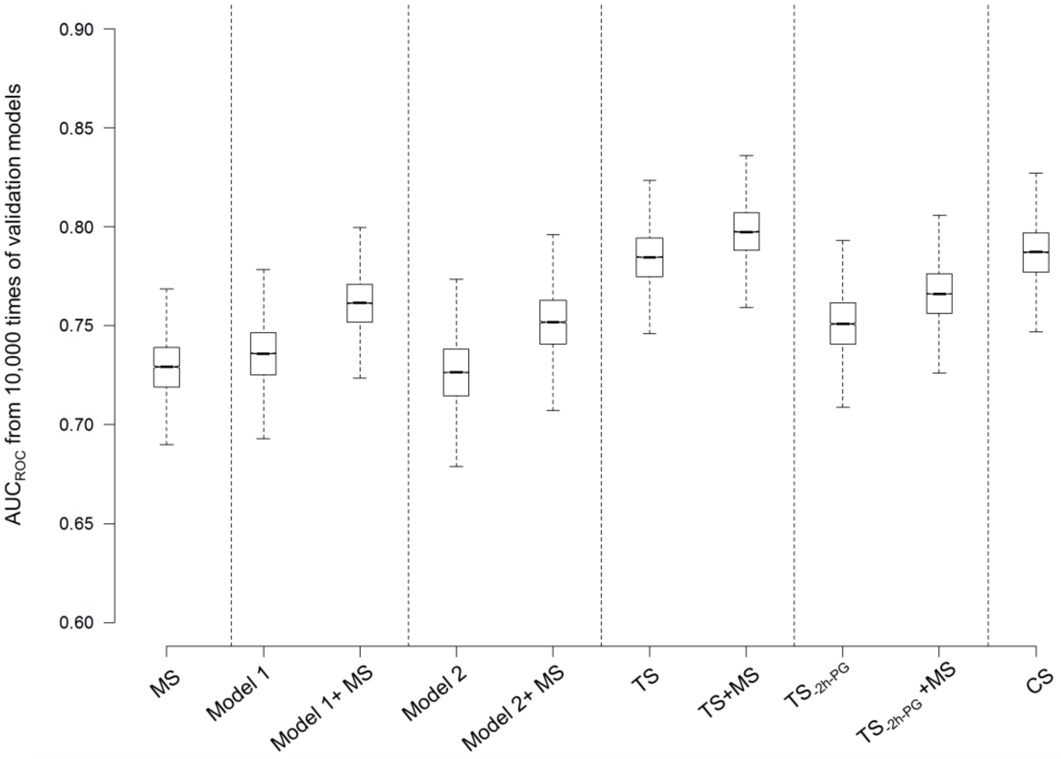

ESM Fig. 5. Comparison of the prediction performance of clinical risk factors, metabolites and their combinations for risk of type 2 diabetes. Comparisons were carried out for predefined model 1, model 2, and optimal selected traditional risk score TS and TS$_{-2h-PG}$, metabolite score (MS), and their combinations, as well as combined score (CS), for risk of type 2 diabetes. Prediction performance of different models trained from metabolites, traditional risk factors and their combinations. The AUC of receiver operation characteristics curves (AUC$_{ROC}$) were obtained from 10,000 models where the samples were randomly splitted into training (60%) and test sets (40%) for prediction and validation; The AUC$_{ROC}$ value was 0.73(95% CI 0.69,0.76) for MS, 0.74(95% CI 0.70,0.77) for model 1, 0.77(95% CI 0.73,0.79) for model 1 + MS, 0.72(95% CI 0.67,0.74) for model 2, 0.75(95% CI 0.72,0.78) for model 2 + MS, 0.78(95% CI 0.76,0.81) for TS, 0.80(95% CI 0.77,0.83) for TS + MS, 0.75(95% CI 0.72,0.78) for TS$_{-2h-PG}$, 0.77(95% CI 0.74,0.79) for TS$_{-2h-PG}$ + MS, 0.79(95% CI 0.76,0.82) for CS. MS included DAG (16:0/18:1), lysoPC (19:1), PC(17:0/18:2), isoleucine, and L-tyrosine (see Fig. 3a). TS consisted of 2 h plasma glucose, fasting plasma glucose, BMI, total cholesterol, triacylglycerols, systolic BP, and red and processed meat intake. CS included FPG, 2h-PG, triacylglycerols, BMI, lysoPC(19:1), lysoPC(18:2), DAG(14:0/18:1), N-acetylglycine, and isoleucine.

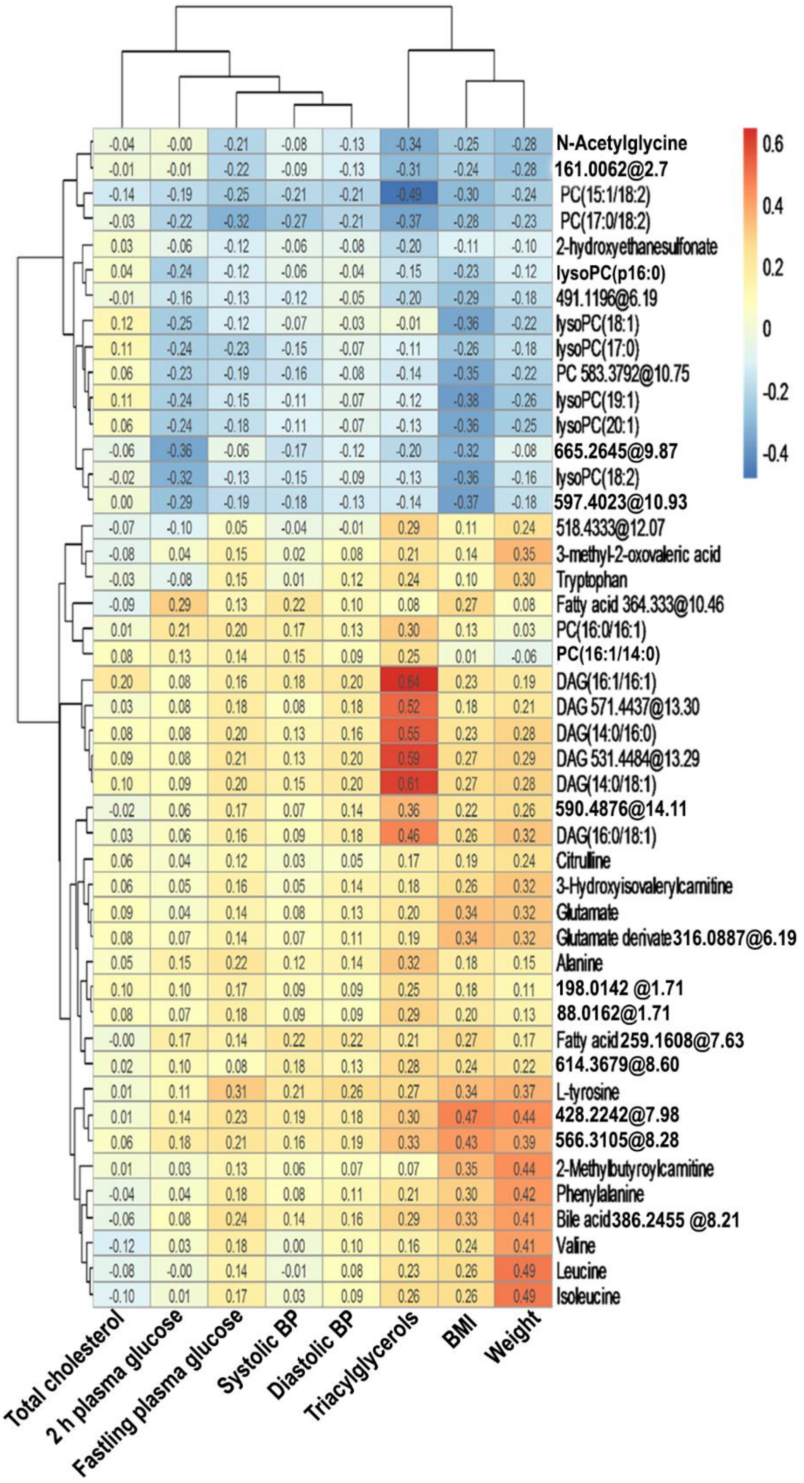

ESM Fig.6 Spearman correlation coefficients between metabolites and traditional type 2 diabetes risk factors among healthy controls.

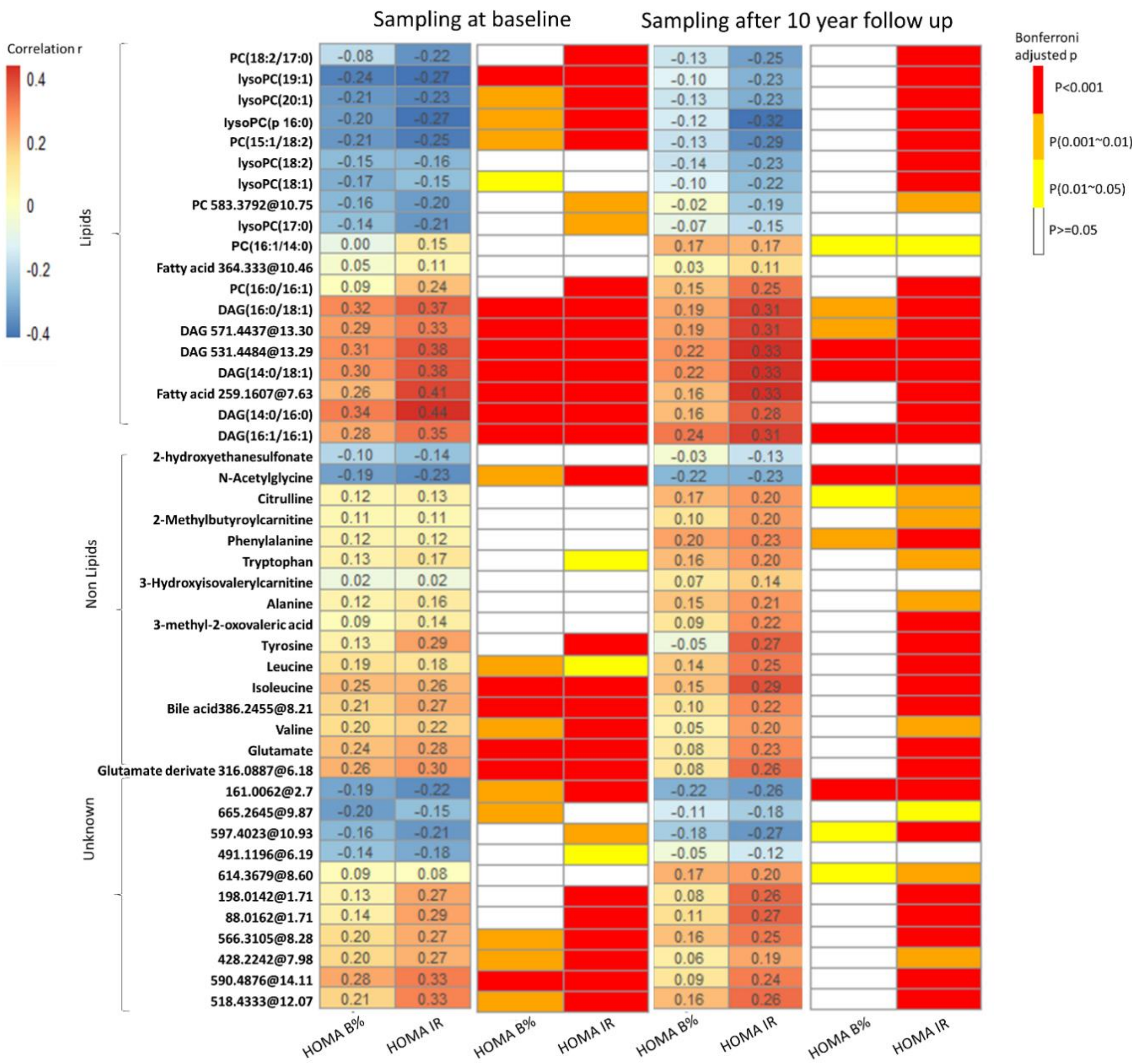

ESM Fig. 7 Partial spearman correlation coefficients between 46 metabolites with HOMA-IR and HOMA-beta cell function (HOMA-B%) at baseline and after 10 year follow up, independent of age, sex, BMI, case/control status.

Reference

1. Norberg M, Wall S, Boman K, et al. The Västerbotten Intervention Programme: background, design and implications. Glob Health Action. 2010,3:1–15.

2. Johansson I, Hallmans G, Wikman A, et al. Validation and calibration of food-frequency questionnaire measurements in the Northern Sweden Health and Disease cohort. Public Health Nutr. 2002,5:487–96.

3. Johansson I, Van Guelpen B, Hultdin J, et al. Validity of food frequency questionnaire estimated intakes of folate and other B vitamins in a region without folic acid fortification. Eur J Clin Nutr . 2010,64:905–13.

4. Brunius C, Shi L, Landberg R. Large-scale untargeted LC-MS metabolomics data correction using between-batch feature alignment and cluster-based within-batch signal intensity drift correction. Metabolomics. 2016,12:173.

5. Hanhineva K, Lankinen MA, Pedret A, et al. Nontargeted Metabolite Profiling Discriminates Diet-Specific Biomarkers for Consumption of Whole Grains, Fatty Fish, and Bilberries in a Randomized Controlled Trial. J Nutr. 2015,145:7–17.

6. Smith C A, Want EJ, O'Maille G, et al. XCMS: processing mass spectrometry data for metabolite profiling using nonlinear peak alignment, matching, and identification. Anal Chem. 2006,78:779–87.

7. Ganna A, Salihovic S, Sundström J, et al. Large-scale Metabolomic Profiling Identifies Novel Biomarkers for Incident Coronary Heart Disease. PLoS Genet. 2014,10:e1004801.

8. Ganna A, Fall T, Salihovic S, et al. Large-scale non-targeted metabolomic profiling in three human population-based studies. Metabolomics. 2016,12:1–13.

9. Smith CA. LC / MS Preprocessing and Analysis with xcms. 2014. 1–13.

10. Stanstrup J, Gerlich M, Dragsted LO, et al. Metabolite profiling and beyond: approaches for the rapid processing and annotation of human blood serum mass spectrometry data. Anal Bioanal Chem. 2013,405:5037–48.

11. Zhu Z-J, Schultz AW, Wang J, et al. Liquid chromatography quadrupole time-of-flight mass spectrometry characterization of metabolites guided by the METLIN database. Nat Protoc. 2013,8:451–60.

12. Libiseller G, Dvorzak M, Kleb U, et al. IPO: a tool for automated optimization of XCMS parameters. BMC Bioinformatics. 2015,16:1–10.

13. Drogan D, Dunn WB, Lin W, et al. Untargeted Metabolic Profiling Identifies Altered Serum Metabolites of Type 2 Diabetes Mellitus in a Prospective, Nested Case-Control Study. Clin Chem. 2014,497:487–97.
